# Direct detection of the chloride release and uptake reactions of *Natronomonas pharaonis* halorhodopsin

**DOI:** 10.1016/j.jbc.2024.107712

**Published:** 2024-08-22

**Authors:** Chihaya Hamada, Keisuke Murabe, Takashi Tsukamoto, Takashi Kikukawa

**Affiliations:** 1 Graduate School of Life Science, Hokkaido University, Sapporo, Japan; 2 Faculty of Advanced Life Science, Hokkaido University, Sapporo, Japan

**Keywords:** membrane transport, chloride transport, chloride pump, photobiology, rhodopsin, ion pump, retinal proteins

## Abstract

Membrane transport proteins undergo multistep conformational changes to fulfill the transport of substrates across biological membranes. Substrate release and uptake are the most important events of these multistep reactions that accompany significant conformational changes. Thus, their relevant structural intermediates should be identified to better understand the molecular mechanism. However, their identifications have not been achieved for most transporters due to the difficulty of detecting the intermediates. Herein, we report the success of these identifications for a light-driven chloride transporter halorhodopsin (HR). We compared the time course of two flash-induced signals during a single transport cycle. One is a potential change of Cl^−^-selective membrane, which enabled us to detect tiny Cl^−^-concentration changes due to the Cl^−^ release and the subsequent Cl^−^-uptake reactions by HR. The other is the absorbance change of HR reflecting the sequential formations and decays of structural intermediates. Their comparison revealed not only the intermediates associated with the key reactions but also the presence of two additional Cl^−^-binding sites on the Cl^−^-transport pathways. The subsequent mutation studies identified one of the sites locating the protein surface on the releasing side. Thus, this determination also clarified the Cl^−^-transport pathway from the initial binding site until the release to the medium.

The cell membrane is a vital reaction site where the cellular uptake and release of various substances are constantly occurring. Active membrane transporters are a class of proteins responsible for the traffic of substances. In the resting state, these proteins do not allow solutes to pass through their interiors. However, once activated, they cause stepwise conformational changes to transport substrates one-way. Out of these multiple steps, substrate release and uptake require substantially large conformational changes, where the protein lumen is alternatively exposed from one side of the membrane to the other (1). Thus, the structural intermediates for these two steps should be identified to better understand the transport mechanism. However, most transporters have not achieved these identifications because of the difficulty in detecting the intermediates themselves. In this study, we report the success of these identifications for the light-driven Cl^−^-transport protein halorhodopsin (HR), a member of the microbial rhodopsin family. For the key experiment, we made a Cl^−^-selective membrane, which allowed us to detect tiny Cl^−^-concentration changes due to Cl^−^ release and the subsequent uptake by HR during its single transport reaction.

Rhodopsins are ubiquitous membrane proteins enabling host organisms to utilize sunlight as energy and information sources (2). In the animal world, they mainly act as light sensors. In contrast, they are functionally diversified in the microbial world (3, 4, 5). Some members act as light sensors similar to animal rhodopsin. However, most act as ion pumps, which actively transport specific ions, such as H^+^, Cl^−^, and Na^+^.

HR is an inward Cl^-^pump from highly halophilic archaeon (6, 7) (Fig. 1*A*). The physiological significance of HR has not been fully resolved. However, many researchers have studied its mechanism as a model transporter because of its advantage in being activated by light (8, 9). As common to other microbial rhodopsin, HR binds the chromophore retinal to the seventh helix *via* a protonated Schiff base linkage (Fig. 1*A*). Upon light illumination, retinal isomerizes from all-*trans* to 13-*cis* state, which resultantly elevates the energy of the protein. This energized state thermally relaxes to the original state through several structural intermediates. During this cyclic reaction called photocycle, HR pumps one Cl^−^ from outside to inside the cell. Figure 1*B* shows a widely accepted photocycle scheme of representative HR from an archaeon *Natronomonas pharaonis* (abbreviated NpHR hereafter) (8, 9). As shown in Figure 1*A*, unphotolyzed NpHR already binds Cl^−^ in the extracellular (EC) side vicinity of the protonated Schiff base. Upon retinal isomerization, Cl^−^ moves to the cytoplasmic (CP) side vicinity of the Schiff base during the L2→N intermediate transition (10). The subsequent formation and decay of O intermediate (N→O and O→NpHR′) have been assigned to the Cl^−^ release and uptake steps at the CP and EC sides, respectively (Fig. 1, *A* and *B*). This transport scheme was supported by previous studies such as detailed kinetic analyses of the intermediate formations (11), electrogenicity during the photocycle (12, 13), transient grating signal due to Cl^−^ release and uptake (14), transient spectral changes in carotenoid bound to NpHR (15), and so on. However, no direct evidence has been reported so far.

**Figure 1:**
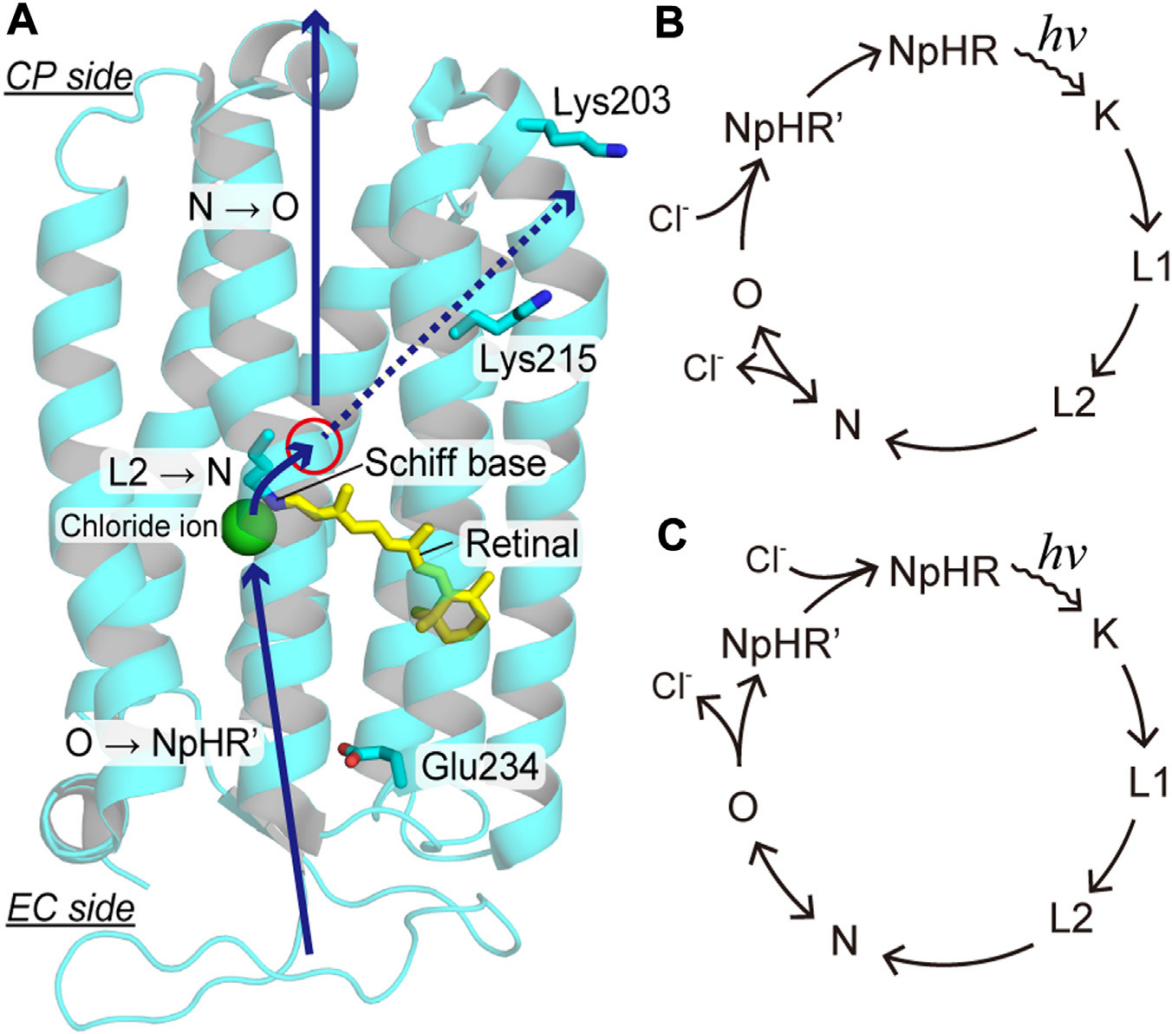
**Overview of the Cl**^**−**^**translocation of HR**. *A*, overall structure of NpHR (PDB code: 3A7K). Unphotolyzed NpHR binds Cl^−^ on the EC side near the protonated Schiff base. During the formation of the N intermediate (L2→N transition), Cl^−^ moves to a position indicated with a *red* circle. For subsequent reactions, it is believed that Cl^−^ is released during O formation (N→O), and then new Cl^−^ is captured during NpHR′ formation (O→NpHR′). These Cl^−^-transfer reactions are schematically shown with solid arrows, but those pathways have not been identified experimentally. The present study shows that Cl^−^ moves diagonally within the CP side and is released from the region between Lys203 and Lys215 residues. This pathway is schematically shown with a broken arrow. *B*, a typical photocycle scheme based on previous studies (for details, see the text). The N and O intermediates make a quasi-equilibrium and are connected with a double-headed arrow. *C*, a photocycle scheme based on the present study, where all data are obtained at Cl^−^ concentrations below 100 mM. The term of "*hν*" in (*B* and *C*) indicates the light stimulation of NpHR.

A short laser pulse can instantaneously activate ion-pumping rhodopsins. Thus, the formation and decay of each intermediate can be analyzed by the time-resolved recordings of specific signals. Due to this advantage, the mechanisms of ion-pumping rhodopsins have been extensively studied as the "model" transport proteins. However, except for H^+^-pumping rhodopsin, it is still difficult to identify the intermediates for substrate release and uptake reactions. These reactions accompany the increase and decrease of substrate concentration in the bulk solution. Thus, their timings can be identified by detecting the flash-induced changes in substrate concentration. Ordinarily, this change is minimal (typically <1 μM), reflecting the concentration of ion-pumping rhodopsin in the aqueous sample. For H^+^-pumping rhodopsin, this tiny change in H^+^ concentration can be detected using pH-sensitive dyes such as pyranine (16, 17), because these dyes have a very small dissociation constant for H^+^ (∼0.05 μM) (18). However, this method does not apply to Na^+^- and Cl^−^- pumping rhodopsins. The sensitive dyes for these ions are also commercially available, but their sensitivities are substantially low (18). Na^+^ or Cl^−^ concentration change of at least a few mM is necessary to cause spectral modifications. Thus, to detect ion transfer reactions by these dyes, we need to concentrate ion-pumping rhodopsin up to an unusually high level and activate it at exceptionally high efficiency. However, in the previous study, we determined the intermediates associated with Na^+^ release and uptake reactions of Na^+^-pumping rhodopsin (19). In that experiment, we utilized a Na^+^-selective membrane instead of Na^+^-sensitive dye and detected the tiny changes in Na^+^ concentration as transient changes in membrane potential. In the present study, we made a Cl^−^-selective membrane for the first time and applied it to the Cl^—^pump NpHR. Thus, we report the first experimental identifications of the relevant intermediates with Cl^−^ release and uptake reactions. The results revealed that these reactions were associated with the last intermediate of NpHR′ (Fig. 1*C*) but not the previously predicted O intermediate (Fig. 1*B*). Furthermore, two additional Cl^−^-binding sites exist on its transport pathway. Subsequent analyses identified one of the sites on the protein surface in the Cl^−^-release side. Thus, this study also clarified a Cl^−^-releasing pathway from the initial binding site until the release to the CP medium.

For H^+^ pump rhodopsin, H^+^ is transported along the transmembrane helix on the CP side. Similarly, in Figure 1*A*, the Cl^−^-transport pathway is schematically shown with a vertical solid arrow (N→O). However, our study revealed that Cl^−^ is transported diagonally, whose pathway is shown with a broken arrow in Figure 1*A*.

## Results and discussion

### Responses of the bare Cl^−^-selective membrane

When two solutions are separated with the Cl^−^-selective membrane (Fig. S1*A*), the partitioning of Cl^−^ occurs at both membrane/solution interfaces and resultantly produces the respective phase-boundary potentials. Chloride ions are equally distributed throughout the membrane as the ionophore-Cl^−^ complex. Thus, the potential gradient does not exist inside the membrane. Consequently, the membrane potential is equal to the sum of two phase-boundary potentials and described by the following Nernst equation (20):

(1) $$\Psi \left(mV\right)=59.2\;\log\;\left[Cl^{-}\right]\;/\;{\left[Cl^{-}\right]}_{ref}\left(25.0\;{}^{\circ}C\right)$$

where *Ψ* stands for the potential difference between "sample" and "reference" media, and [Cl^−^] and [Cl^−^]_ref_ represent the Cl^−^ concentrations in these mediums, respectively.

**Figure 2:**
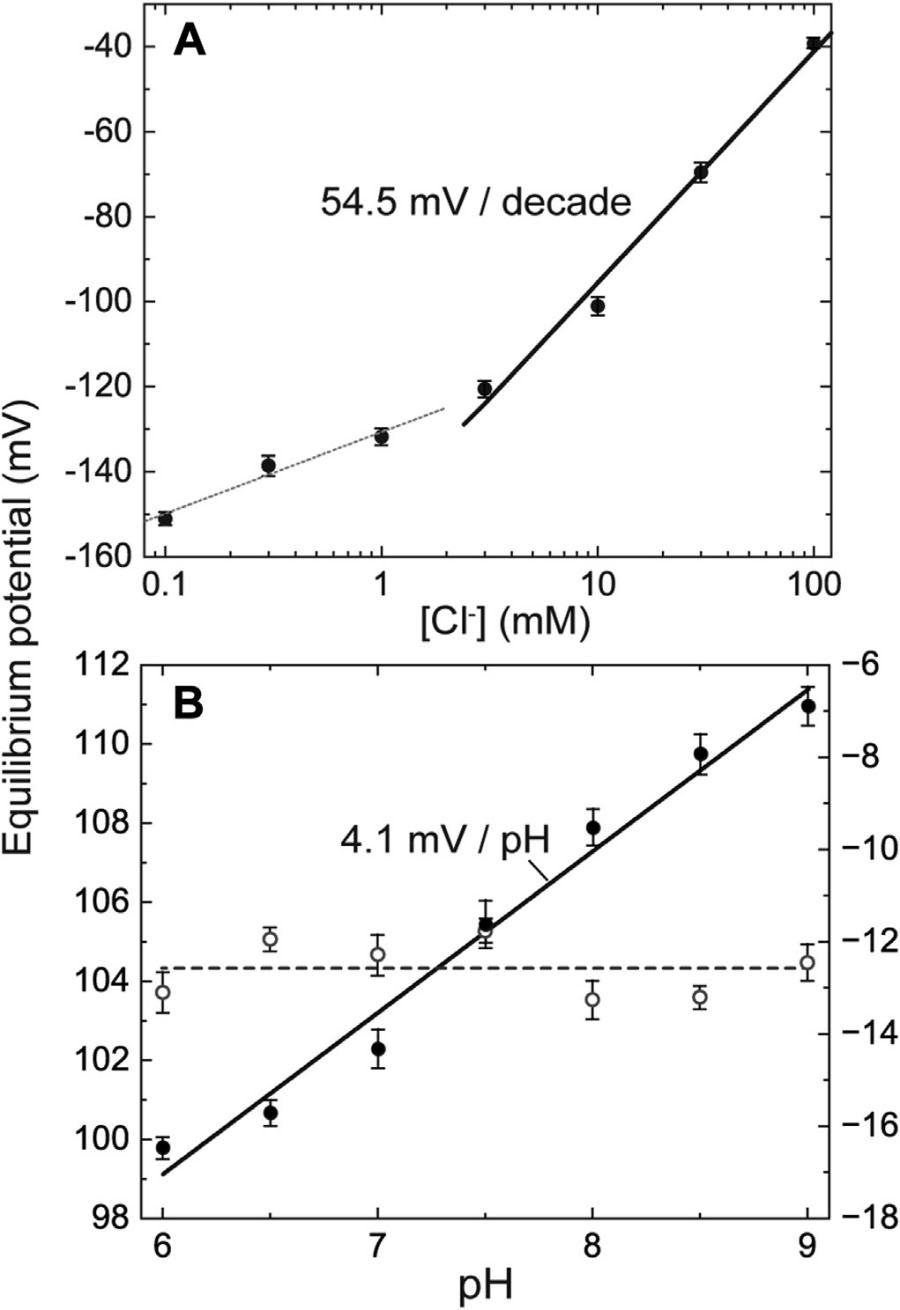
**Basic characterization of a bare Cl**^**−**^**-selective membrane**. *A*, the response against Cl^−^ concentration. The buffer pH was kept at 7.0. At lower Cl^−^ concentration, the slope of the plot is only 19.1 mV/decade, probably reflecting a smaller amount of the ionophore-Cl^−^ complex within the membrane. But, above 3 mM, the slope is close to the ideal value (59.2 mV/decade). *B*, the response against pH. The Cl^−^-selective membrane exhibited small but distinctive pH dependence (*closed circles*) with a slope of 4.1 mV/pH. The open circles plotted with the right axis are the data obtained with a membrane that did not contain Cl^−^ ionophore. The Cl^−^ concentration was maintained at 1 M. In (*A* and *B*), all data are presented as the mean ± SD (n = 3).

First, we tested the response of a bare Cl^−^-selective membrane. The result is shown in Figure 2*A*, where the membrane potential is plotted against the Cl^−^ concentration in the sample medium while maintaining it at 1 M for the reference medium. Above 3 mM Cl^−^, the slope of the plot is 54.5 mV/decade, which is close to the ideal Nernst slope of 59.2 mV/decade. Below 3 mM, the slope is 19.1 mV/decade. This low sensitivity might reflect the relatively weak affinity between Cl^−^ and the ionophore. As a result, the amount of the ionophore-Cl^−^ complex in the membrane might be smaller at lower Cl^−^ concentrations. The presence of SO_4_^2−^, which is added to the buffer solution to maintain the ionic strength, might also affect the slope. This Cl^−^ ionophore is known to interact with SO_4_^2−^ as well (21). The interaction is much weaker than the interaction with Cl^−^. However, the buffer solutions below 3 mM Cl^−^ contain more than 32 mM SO_4_^2−^. These relatively high concentrations of SO_4_^2−^ might inhibit the formation of the ionophore-Cl^−^ complex.

Unexpectedly, we found that the Cl^−^-selective membrane also senses the pH, as shown in Figure 2*B* (closed circles), where both media contain 1-M Cl^−^ and the pH of the reference medium is kept at 7. As indicated by the open circles, the PVC membrane without the Cl^−^ ionophore exhibits no pH dependence of the membrane potential. Thus, this Cl^−^ ionophore probably interacts with H^+^, although the detailed mechanism remains unknown. Due to this pH sensitivity, light-induced potential change should also occur if NpHR exhibits an H^+^ transfer reaction during the photocycle.

### Cl^−^-concentration change by NpHR under 1-s illumination

**Figure 3:**
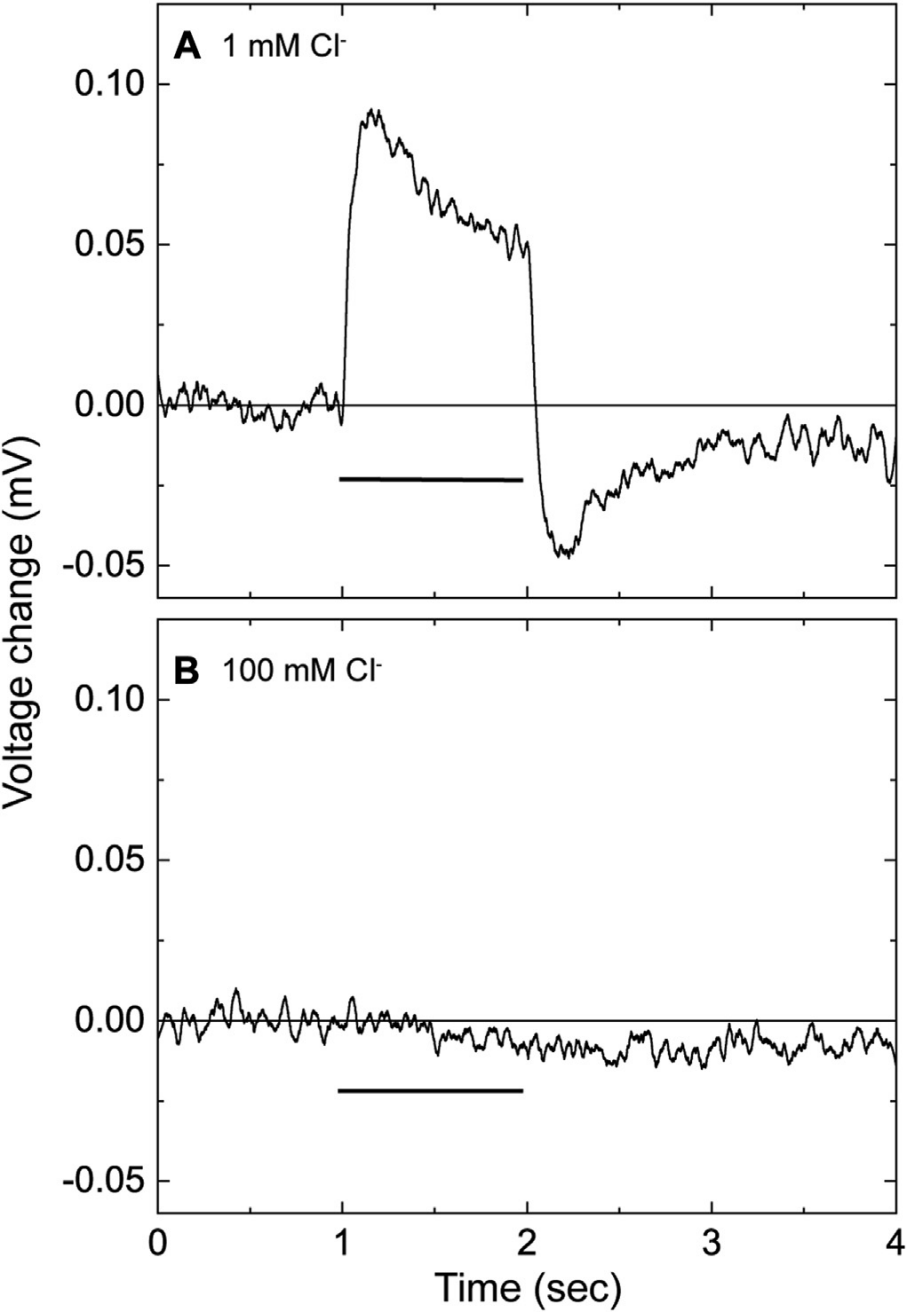
**Potential change of the Cl**^**−**^**-selective membrane under constant illumination**. The lipid-reconstituted NpHR covered the membrane surface. The horizontal bar indicates the duration of illumination. The Cl^−^ concentration in the sample medium was 1 mM (*A*) and 100 mM (*B*).

Next, we tried to detect the Cl^−^ concentration change induced by NpHR. Here, we deposited the lipid-reconstituted NpHR on one side of the Cl^−^-selective membrane and then illuminated it for 1 s. As shown in Figure 3*A*, illumination induces a positive potential change in the presence of 1-mM Cl^−^. The deposited NpHR faces the sample medium (Fig. S1*A*). Thus, the positive potential change reflects increasing Cl^−^ concentration, indicating that the photolyzed NpHR releases Cl^−^ first, followed by uptake as previously considered. With the increase in Cl^−^ concentration of the sample medium, the potential change becomes small and finally disappears at around 100-mM Cl^−^ (Figs. 3*B* and S2). This decrease can be explained by the following equation:

(2) ΔΨ(mV)=k.log(1+Δ[Cl−]/[Cl−])

where *k* denotes the slope of Figure 2*A*, Δ[Cl^−^] stands for the light-induced Cl^−^ concentration change by NpHR, and Δ*Ψ* indicates the resultantly measured potential change. The above equation indicates that Δ*Ψ* depends on Δ[Cl^−^] and [Cl^−^], the Cl^−^ concentration in the bulk medium. Thus, at higher Cl^−^ concentrations, the ratio of Δ[Cl^−^]/[Cl^−^] becomes almost zero, and consequently, Δ*Ψ* also becomes zero, although NpHR causes the Cl^−^ concentration to change (Δ[Cl^−^]). In other words, the disappearance of Δ*Ψ* confirms that the observed potential change reflects only the change in Cl^−^ concentration.

In subsequent experiments using laser pulses, we employed the Cl^−^ concentrations above 1 mM. As shown in Figure 2*A*, at 1 mM Cl^−^, the sensitivity of the Cl^−^-selective membrane is low. However, based on the relationship in Equation 2, relatively large Cl^−^-transfer signals were observed at 1 mM Cl^−^, as shown below. The low sensitivity should not distort the time course of the potential change. Thus, the data at 1 mM Cl^−^ is also available to know the Cl^−^ transfer timings during the photocycle.

### Cl^−^-concentration change by NpHR during a single photocycle

**Figure 4:**
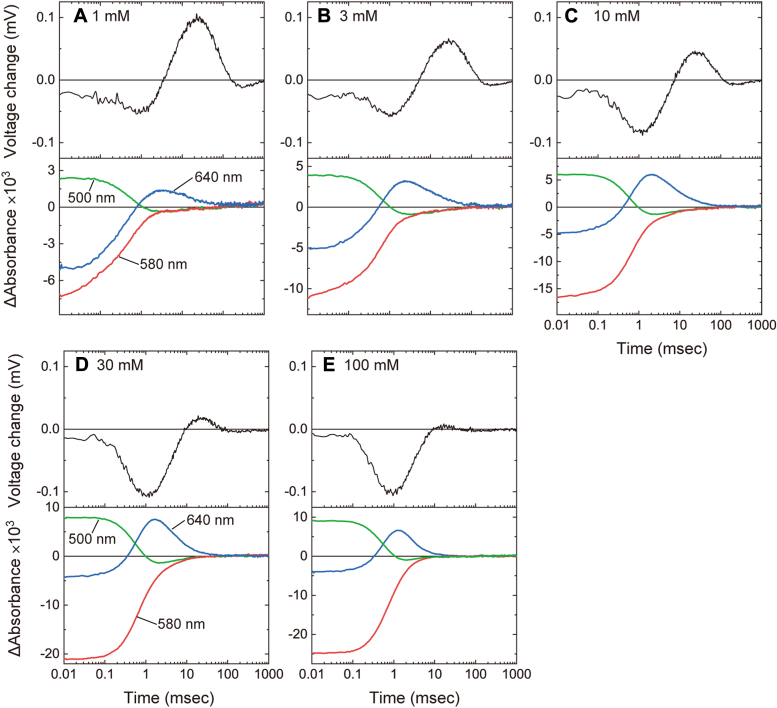
**The time course of two flash-induced signals with various Cl**^**−**^**concentrations**. The flash-induced potential changes in the *upper panels* were measured for the lipid-reconstituted NpHR deposited on the Cl^−^-selective membrane. We measured the flash-induced absorption changes in the *lower panels* for the lipid-reconstituted NpHR immobilized in the acrylamide gels. The Cl^−^ concentrations are indicated in the *upper panels*.

Next, we tried to detect the Cl^−^-concentration change during the single photocycle. Herein, we used the 5-ns laser pulse to activate NpHR. In Figure 4, the measured potential changes at various Cl^−^ concentrations are plotted in the upper panels, and the corresponding flash-induced absorption changes are plotted in the lower panels. Regarding the potential change (upper panels), we obtained unexpected results. Besides the positive potential change, the preceding negative potential change is also observed. The magnitude of the positive potential change gradually decreases with the increase in Cl^−^ concentration and finally disappears (Fig. 4, *A*–*E*), similar to the potential change under 1-s illumination (Figs. 3 and S2). Thus, a positive signal should reflect the Cl^−^-concentration change by NpHR, that is, NpHR exhibits Cl^−^ release first followed by uptake.

In contrast to the positive signal, the preceding negative peak around 1 ms constantly increases with the Cl^−^ concentration (Fig. 4, *A*–*E*). Thus, this signal does not originate from the Cl^−^ concentration change. Unphotolyzed NpHR binds Cl^−^ with a dissociation constant of about several mM (15, 22), and this Cl^−^ binding is requisite to exhibit Cl^−^-pumping photocycle. The amount of Cl^−^-pumping NpHR increases at higher Cl^−^ concentration. This increase relates to an increasing negative peak, that is, the negative signal originates from the events during the Cl^−^-pumping photocycle. Only the positive signal is observed for the potential change by 1-s illumination (Fig. 3*A*). This result indicates that under 1-s illumination, the intermediate associated with Cl^−^ release mainly accumulates due to its slow decay. In contrast, the preceding intermediate related to the negative potential change accumulates only slightly due to its fast decay. Thus, the negative potential change is not observed. The origin of the negative potential change will be discussed later.

The flash-induced absorbance changes reflect the time-dependent formation and decay of intermediates and the recovery of the original dark state. In the lower panels of Figure 4, the absorbance changes at three typical wavelengths are plotted. The sample was the lipid-reconstituted NpHR immobilized in the acrylamide gel to avoid precipitation. In the dark state, Cl^−^-bound NpHR has an absorption maximum of 580 nm. Thus, the negative absorption change at 580 nm reflects the depression of the original state and its subsequent recovery due to the end of the photocycle. In contrast, the positive signal at 500 nm reflects the formation of the L1 and L2 intermediates (Fig. 1, *B* and *C*), which have almost the same absorption spectra. The formation of L1 is very fast and completes within 10 μs. Thus, our data reflect the photocycle after the L1 formation. The N intermediate after L2 (Fig. 1, *B* and *C*) also has an absorption at 500 nm. However, its accumulation is known to be small at low Cl^−^ concentrations (<100 mM) (15, 23). Thus, the decay of the 500 nm signal indicates the decay of L2. At 640 nm, the O intermediate appears as the positive signal. As mentioned above, O has been assigned to the Cl^−^-releasing state. If this is the case, the positive potential changes in upper panels should overlap with the accumulation of O intermediate. However, the potential change seems to appear after the O accumulation, suggesting that Cl^−^ release and uptake are associated with the next intermediate NpHR′ (Fig. 1, *B* and *C*), which is not clearly observed by the flash-induced absorbance change as described below. Instead of the positive potential change, O accumulation seems to overlap with the preceding negative potential change. We doubted that NpHR on the Cl^−^-selective membrane might exhibit altered photocycle kinetics. Thus, we also measured the flash-induced absorbance changes for NpHR deposited on the Cl^−^-selective membrane. The data are shown in Fig. S3, together with the membrane potential changes, which are the same data in Figure 4. The time courses of absorption changes are essentially the same as those of NpHR in the acrylamide gel. Even on the Cl^−^-selective membrane, the O intermediate accumulates at almost the same time as the negative potential change and decays before the positive potential change.

### Detection of the H^+^-transfer reactions by NpHR

As mentioned above, the Cl^−^-selective membrane senses not only Cl^−^ but also H^+^. Thus, the negative potential change in Figure 4 might reflect the H^+^-transfer reactions of NpHR. We tested this hypothesis using the ITO electrode, which allows us to detect H^+^ transfer reactions during a single photocycle (24). The experimental results are summarized in Supplemental Results 1-1. The obtained data surely indicated that the negative peak of potential change originated from the H^+^ release and the subsequent uptake reactions by NpHR. Thus, NpHR exhibits H^+^-transfer reactions before the Cl^−^-transfer reactions.

In the upper panels of Figure 4, a slight negative potential change is already present at 0.01 ms and keeps an almost constant value until 0.1 ms. In the ITO data, the corresponding voltage change does not appear (Fig. S4, *A* and *B*). Thus, the early negative potential change does not seem to originate from the H^+^-transfer reactions. By contrast, this potential change remains at 100-mM Cl^−^ and thus cannot be assigned to the Cl^−^-transfer reactions. At present, we cannot propose a rational origin for this potential change.

### Detailed analyses of the flash-induced absorbance changes

**Figure 5:**
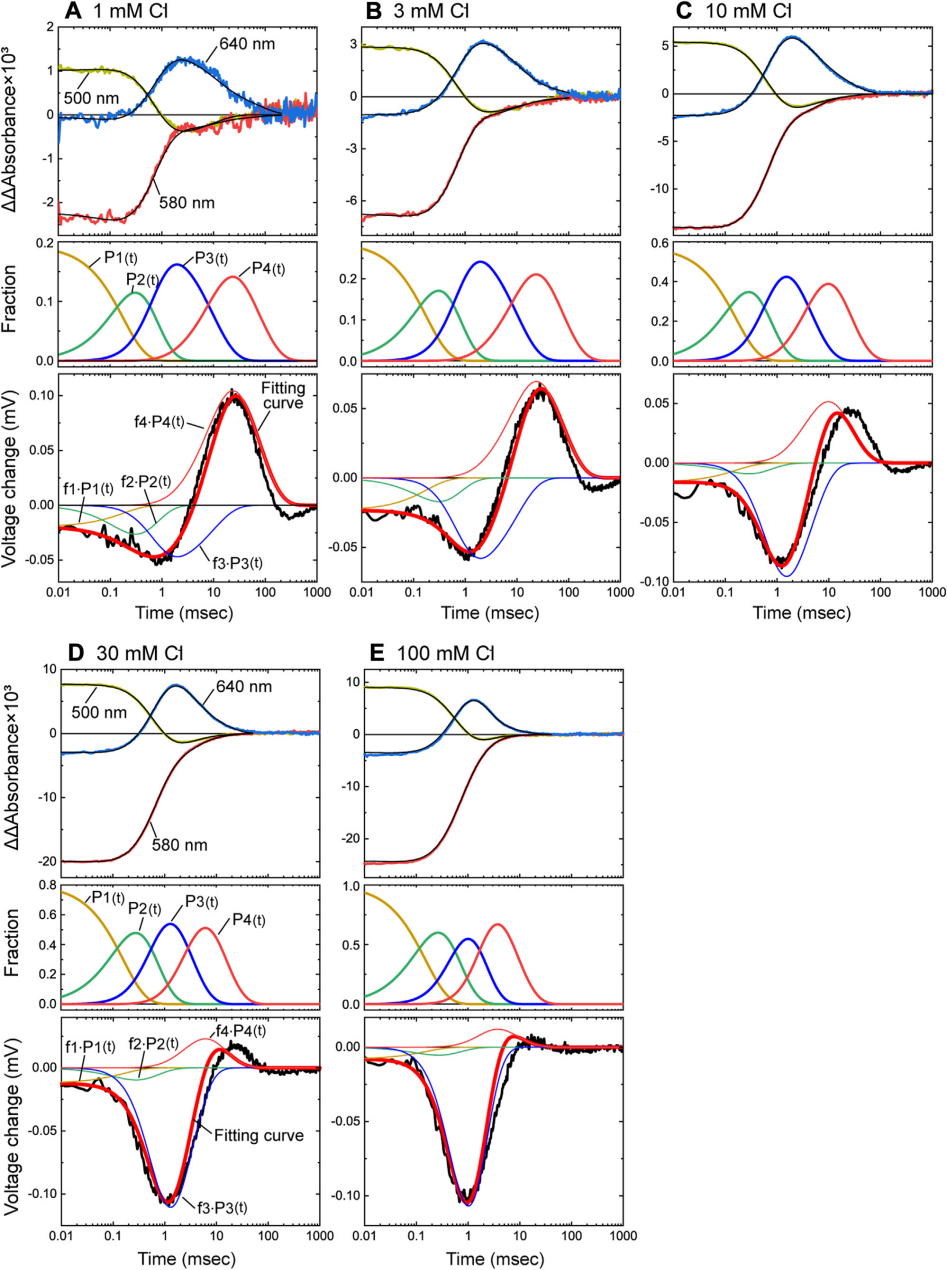
**Comparisons between the timings of intermediate accumulations and the membrane potential changes**. The *top panels* indicate the flash-induced absorbance changes caused by the Cl^−^-pumping NpHR (*thick lines*) and their best-fit curves (*thin black lines*). Only data at three wavelengths are shown. The data shown in *thick lines* are the same as the data in *thick lines* in Fig. S6, *B*–*F*. The panels in the *second rows* indicate the time course of fractions of Pi (i = 1–4) states (*P*_i_(*t*)). They were calculated using the determined decay time constants of Pi states, which are summarized in Fig. S7. The relative magnitudes of each fraction (*P*_i_(*t*)) were adjusted so that the *P*_1_(0) is equal to the *f*_Cl_, which represents the fraction of the Cl^−^-bound NpHR in the *dark state*. The *bottom panels* involve the raw data of membrane potential change (*thick black lines*), which are the same as those in the upper panels of Figure 4. The thick red lines are the simulation curves calculated by Equation 3, whose components of *f*_i_⋅*P*_i_(*t*) are shown with *thin lines*. For details, see the text.

**Figure 6:**
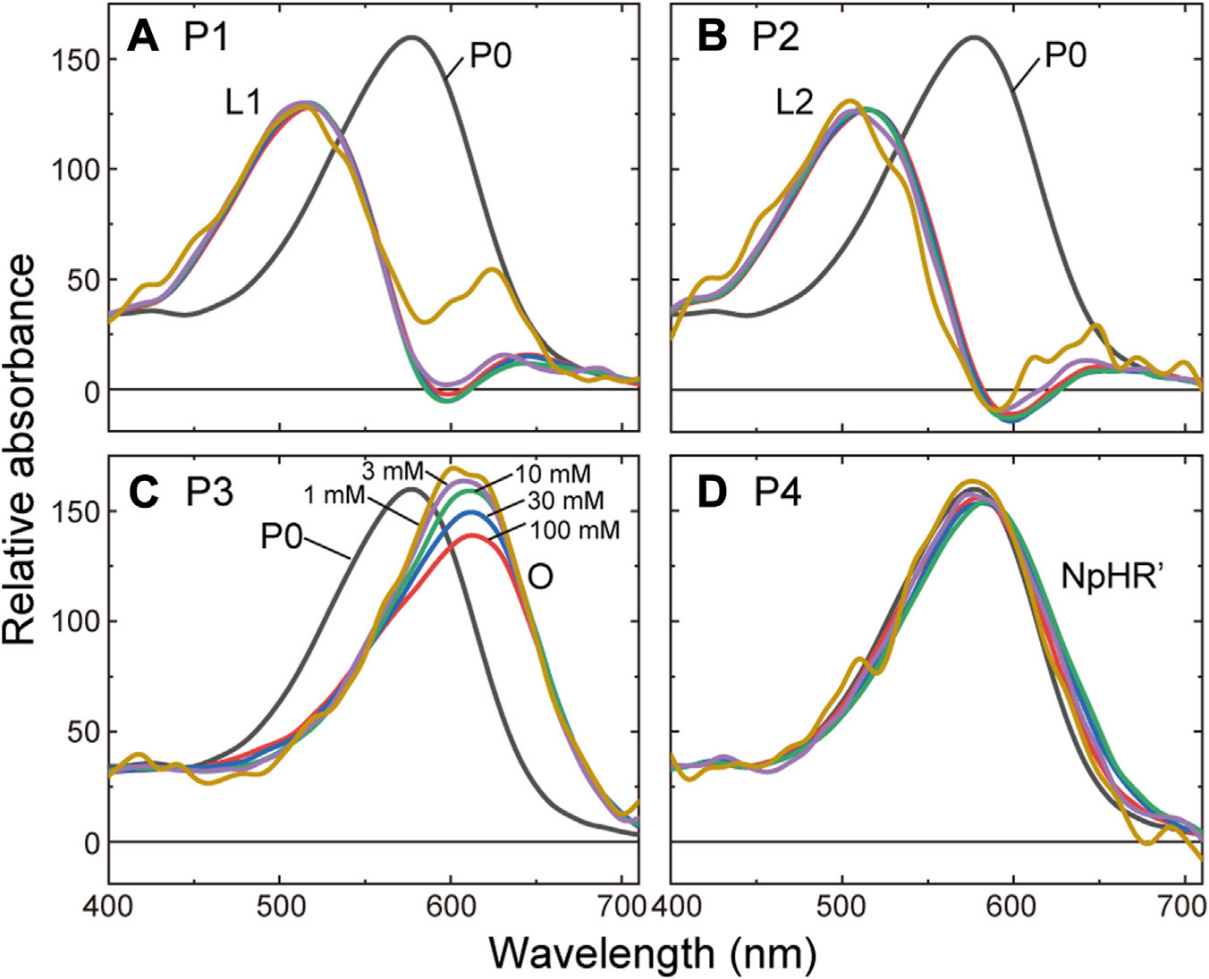
**Absolute spectra of Pi (i = 1–4) states**. The *thick gray line* indicates the spectrum of the P0 state. The Pi spectra at 1 to 100 mM were calculated and simultaneously shown in each panel. The Cl^−^ concentrations are indicated in (*C*).

Figure 4 suggests that NpHR′ is associated with Cl^−^ release and uptake events. However, NpHR′ has nearly the same absorption spectrum as the dark state, so its formation does not clearly appear in the absorption changes. Thus, we performed global fitting analyses for all wavelength data from 400 to 710 nm (at a 10-nm interval) to estimate the accumulation timing of NpHR′ because a larger quantity of wavelength data probably increases the estimation accuracy. In this study, we used the sequential irreversible model containing four kinetically distinguishable states (P1–P4) after previous studies (15, 23). The current problem is the involvement of Cl^−^-free NpHR in the sample. As mentioned above, the dissociation constant (*K*_d_) of NpHR for Cl^−^ is several mM. Thus, the sample involved a significant amount of Cl^−^-free NpHR at a lower Cl^−^ concentration. The Cl^−^-free NpHR exhibits a “nonpumping photocycle” after photoactivation and causes absorption changes. Thus, before the global fitting analysis, we removed the contribution of "nonpumping photocycle" from the measured absorbance changes. The procedures are summarized in Supplemental Results 1–2. The top panels of Figure 5 involves three wavelength data after the procedures (thick lines). We performed the global fitting analyses against these data. The resultant best-fit curves are also plotted in the top panels of Figure 5 (thin black lines) and the decay time constants of Pi states are summarized in Fig. S7. Using the determined parameters, we calculated the absorption spectra of Pi states, summarized in Figure 6. The obtained spectra are consistent with previous studies (15, 23). The P1 and P2 states (Fig. 6, *A* and *B*) have almost similar spectra with λ_max_ at around 520 nm and thus are assigned to the L1 and L2 intermediates, respectively. Only the P3 states (Fig. 6*C*) have the redshifted spectra, which correspond to O. At higher Cl^−^ concentration, the preceding N is known to also appear in the P3 state by making the quasi-equilibrium with O. In Figure 6*C*, the amplitude of O exhibits a slight decrease with increasing Cl^−^ concentration, which reflects the shift of equilibrium toward N, although the appearance of N at around 500 nm is still unclear. The next P4 states (Fig. 6*D*) have almost the same spectra as the dark state and are assigned to NpHR′. Thus, the accumulation timings of P3 and P4 surely reflect the accumulation timings of O and NpHR′, respectively. The next concern is whether these timings overlap with negative (H^+^ transfer) and positive (Cl^−^ transfer) potential changes.

### Detailed comparisons between the timings of intermediate accumulations and the membrane potential changes

The decay time constants of the P1–P4 states were already determined (Fig. S7). Thus, we calculated the time course of their fractions, referred to with *P*_i_(*t*) (i = 1–4), and plotted in the second rows of Figure 5. In the bottom panels, the potential changes are plotted again with thick black lines. As mentioned above, even in the early time range (<0.1 ms), the membrane potential has a small negative change, but we now focus on the latter part associated with the P3 and P4 states. Regarding the negative potential changes, their time courses almost match the respective *P*_3_(*t*), especially above 3-mM Cl^−^ (Fig. 5, *B*–*E*). Thus, the H^+^-transfer reaction seems to be associated with the P3 state. How about the positive potential change? At 1-mM and 3-mM Cl^−^ (Fig. 5, *A* and *B*), the time courses of positive potential changes closely match the respective *P*_4_(*t*), where the peak positions of the two signals are almost similar. At higher Cl^−^ concentration (Fig. 5, *C*–*E*), however, the peak position of *P*_4_(*t*) becomes faster than that of positive potential change. Thus, P4 accumulation seemingly occurs before the Cl^−^-transfer reactions. However, this mismatch might occur by the following two kinds of Cl^−^-dependent changes: At higher Cl^−^ concentration, (a) the peak positions of *P*_3_(*t*) and *P*_4_(*t*) become closer, and (b) "net" potential change due to Cl^−^ transfer becomes smaller according to Equation 2. Resultantly, "true" positive potential change might be covered by the preceding negative potential change, and then only the last part of positive potential change might remain.

If the H^+^ and Cl^−^-transfer reactions associated with the P3 and P4 states, the observed potential change should have the shape of "*f*_3_⋅*P*_3_(*t*) + *f*_4_⋅*P*_4_(*t*)," where *f*_3_ and *f*_4_ are the negative and positive coefficients, respectively, to adjust the magnitudes of respective peaks. Here, we tried to simulate the overall potential change by the following equation:

(3) $$f_{1}.P_{1}\left(t\right)+f_{2}.P_{2}\left(t\right)+f_{3}.P_{3}\left(t\right)+f_{4}.P_{4}\left(t\right)$$

The first two terms are to simulate the negative potential change in the early time range. Thus, *f*_1_ and *f*_2_ have negative values. The simulated curves are shown in the bottom panels in Figure 5 with thick red lines. We initially used fitting software to determine the *f*_1_–*f*_4_ values. However, this method ignored the positive potential change, especially at high Cl^−^ concentrations. Those simulated curves are shown in Fig. S8. Thus, we manually modified those coefficients to further reduce the fitting error in the positive peak area. The resultant simulation curves are plotted in Figure 5. These curves roughly simulate the entire potential changes, indicating that H^+^ and Cl^−^-transfer reactions are associated with O and NpHR′, respectively.

In the bottom panels of Figure 5, *f*_i_⋅*P*_i_(*t*) (i = 1–4) components are also plotted with thin lines. The individual values of *f*_1_–*f*_4_ are summarized in Fig. S9, which indicates that *f*_4_ is inversely proportional to the Cl^−^ concentration. This relationship is theoretically derived from Equation 2 as described in Supplemental Discussion 2-1, indicating that the determined *f*_4_ values also support the association of NpHR′ with the Cl^−^-transfer reaction.

### Presence of additional Cl^−^-binding sites on the Cl^−^-transport pathway

**Figure 7:**
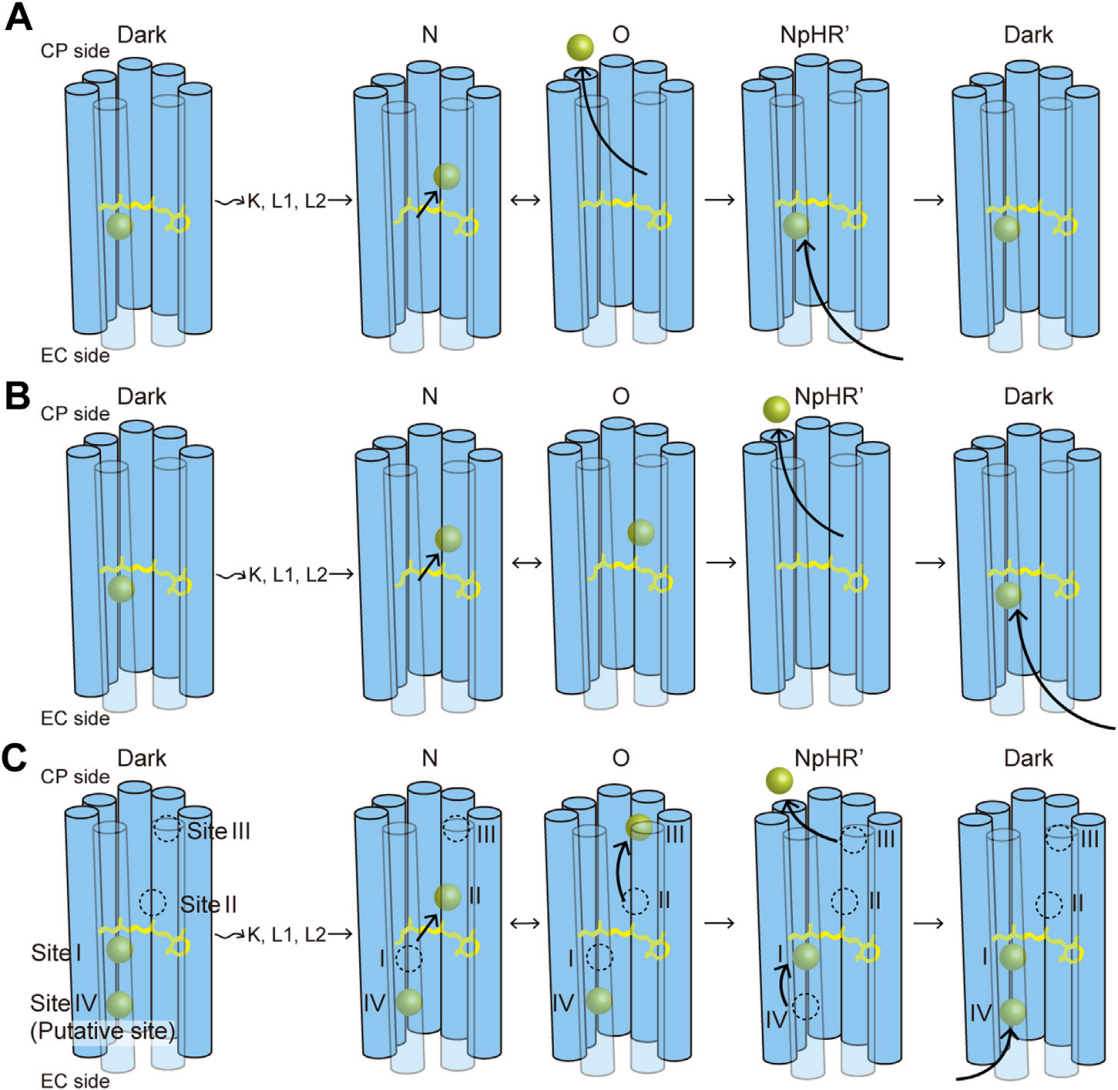
**Cl**^**−**^**-transfer reactions during the NpHR photocycle**. *A*, the previous model assigns Cl^−^ release and uptake to the N→O and O→NpHR′ transitions, respectively. *B*, the modified model simply reflects our experimental results. *C*, the corrected model considers the absorption spectra of O and NpHR′ intermediates.

As mentioned above, O was considered the Cl^−^-releasing intermediate. This photocycle model is schematically shown in Figure 7*A*, where the Cl^−^ release and uptake processes are associated with the N→O and O→NpHR′ transitions, respectively. However, our results oppose this model. As shown in Figure 7*B*, our data indicated that the Cl^−^ release and uptake are associated with O→NpHR′ and NpHR′→NpHR (Dark), respectively. However, Figure 7*B* is inconsistent with the absorption spectra of O and NpHR′. O has the redshifted absorption spectrum, similar to that of Cl^−^-free unphotolyzed NpHR. A previous FTIR study also revealed the close similarity between O and the Cl^−^-free unphotolyzed state (25). Thus, at O, Cl^−^ should not be located near the retinal, but at a binding site far from the retinal, as shown in Figure 7*C*. Namely, an additional Cl^−^-binding site exists on the Cl^−^-releasing pathway. In contrast, NpHR′ has almost the same absorption spectrum as the dark state, indicating that Cl^−^ already binds to the vicinity of the retinal Schiff base, as shown in Figure 7*A*. However, NpHR′ in Figure 7*B* does not yet bind Cl^−^. To avoid this discrepancy, the other Cl^−^-binding site needs to be considered on the uptake pathway, as shown in Figure 7*C*. In summary, our experimental data suggest the photocycle model in Figure 7*C*, where additional two Cl^−^-binding sites are supposed on both the CP and EC sides and two Cl^−^-binding sites on the EC side are supposed to already bind Cl^−^ in the dark state. In Figure 7*C*, four binding sites are labeled from Sites I to IV. Upon activation by light, Cl^−^ at Site I moves to Site II during the formation of the N intermediate. During the formation of the next O, this Cl^−^ moves to Site III, which is far from the retinal and does not affect the absorption spectrum. During the formation of the next NpHR′, this Cl^−^ is released to the CP medium, and simultaneously, the Cl^−^ at Site IV moves to Site I. Reflecting the Cl^−^ binding to Site I, the absorption spectrum becomes almost the same with the dark state. The last step of NpHR′ decay is the Cl^−^ uptake by Site IV from the EC medium.

### Exploration of the additional Cl^−^-binding sites

**Figure 8:**
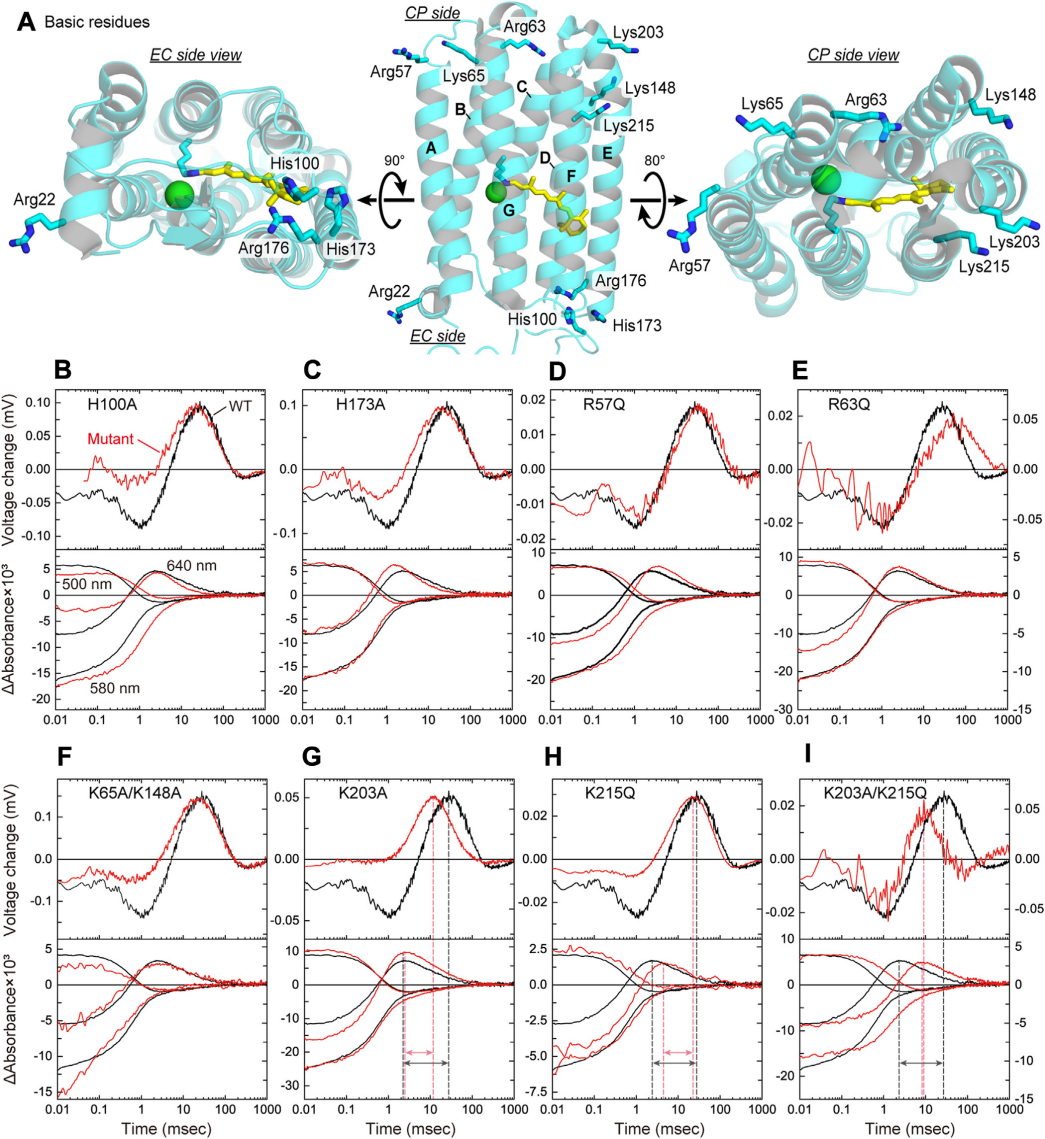
**Explorations of the additional Cl**^**−**^**-binding sites**. *A*, the positions of basic residues around the protein surface. *B*–*I*, comparison of the time course of flash-induced membrane potential changes (*upper panels*) and flash-induced absorbance changes (*lower panels*) at 3 mM Cl^−^. Each panel involves both the data of the respective mutant (*red lines*) and the wild-type NpHR (*black lines*). These *red* and *black lines* are plotted in each panel using *left and right axes*, respectively. Panels *G*–*I* display *vertical lines* that denote the peak positions of potential change and O accumulation. The *double-head arrows* indicate the durations of these peak positions. The data for wild-type NpHR (*black lines*) are the same as those in Figure 4*B*.

Next, we tried to identify the additional Cl^−^-binding sites (Sites III and IV) through amino acid substitutions. These binding sites are probably located around the CP and EC surfaces and involve the positively charged residues as the key components. NpHR has four and six basic residues on EC and CP surfaces, respectively. Their positions are shown in Figure 8*A*. We replaced all of them with neutral residues and measured flash-induced membrane potential changes and flash-induced absorption changes at 3-mM Cl^−^. The results are summarized in Figure 8, *B*–*I*. For EC side mutants, R22Q and R176Q were not expressed in the *Escherichia coli* membrane, and thus their data could not be obtained. The data of H100A and H173A mutants are summarized in Figure 8, *B* and *C*, respectively. In each panel, the data of wild-type NpHR are also plotted (black lines). If the Site IV is removed by the EC side mutation, the timing of Cl^−^ uptake probably becomes faster, that is, Cl^−^ uptake should occur during the NpHR′ formation, which is the same timing as the Cl^−^ release. Thus, the Cl^−^-concentration changes might cancel each other, so the potential change might become small. However, both mutants exhibited distinct potential changes with almost the same timing as wild-type NpHR. Moreover, the time course of absorption changes is also the same between the mutants and the wild-type NpHR. Thus, we failed to identify Site IV and thus its existence is still in question. In response to these results, in Figure 7*C*, Site IV is labeled as "(Putative site)". The additional discussion on the presence of Site IV is described in Supplemental Discussion 2-2.

The data for CP side mutants are shown in Figure 8, *D*–*I*. If Site III is removed by the mutation, the Cl^−^ release probably occurs during the formation of O, which is faster than that of NpHR′ for the wild-type NpHR. Such a faster Cl^−^ release is not observed for R57Q, R63Q, and the double mutant K65A/K148A (Fig. 8, *D*–*F*). NpHR is known to spontaneously assume trimer conformation, where Lys65 and Lys148 residues consist of the Cl^−^-binding site between two protomers (26). The double mutation of K65A/K148A probably removes the Cl^−^-binding ability but does not impact flash-induced data. Similar to these mutants, K203A and K215Q mutants release Cl^−^ after forming the O intermediate (Fig. 8, *G* and *H*). However, the time delay between the "O formation" and "Cl^−^ release" decreases in these mutants. These time delays are schematically shown in the lower panels with double-head arrows, which indicate the time durations between the peak positions of O accumulation and positive potential change. For K203A (Fig. 8*G*), the positive potential change occurs with faster timing compared to the wild-type NpHR, although there are no essential differences in the kinetics of O accumulation. Consequently, the time delay becomes shorter. In contrast, for K215Q (Fig. 8*H*), there is no essential difference in the time course of potential changes, although the formation of O intermediate becomes slower compared to the wild-type NpHR. Thus, the time delay also becomes shorter. As shown in Figure 8*A* (middle and right panels), these lysine residues are located on different helices (E and F), but their spatial distance is relatively close and might collaboratively form Site III. Thus, we made a double mutant K203A/K215Q, whose data are shown in Figure 8*I*. Interestingly, its positive potential change occurs almost simultaneously as the formation and decay of the O intermediate, indicating that the double mutation removes Site III. The amplitude of the positive potential change becomes significantly small compared to the wild-type NpHR, which is reasonable because both Cl^−^ and H^+^ transfer reactions simultaneously occur during the formation and decay of the O intermediate. Thus, their signals cancel each other. In the K203A/K215Q mutant, Cl^−^ is probably released from Site II without getting trapped at Site III.

As mentioned earlier, the removal of Site III should make the timing of Cl^−^ release faster but should not affect the timing of Cl^−^ uptake because the latter is handled by Site IV on the opposite EC side. However, in K203A/K215Q, Cl^−^ uptake also becomes faster, which occurs during O decay and not during NpHR′ decay for the wild-type NpHR. At present, we cannot explain this result. Site IV should be far from Site III, but these sites might have long-distance interaction. A similar interaction is also suggested by data from R63Q mutant (Fig. 8*E*). This mutant has delayed Cl^−^ release and uptake reactions compared to the wild-type NpHR. This residue is located on the CP surface. Thus, its mutation might affect the timing of Cl^−^ release through conformational modifications. However, it does not explain why this mutation would also affect the timing of Cl^−^ uptake on the opposite EC side. Due to a long-distance interaction, the delay in Cl^−^ release might lead to a delay in Cl^−^ uptake.

The mutations of K203 and K215 residues also raise a question regarding the H^+^-transfer reactions. As shown in Figure 8*G*, the H^+^-transfer reactions become negligibly small by the K203A mutation. However, Lys203 is not the H^+^-releasing residue because the H^+^-transfer reaction is also detected in the K203A/K215Q mutant (Fig. 8*I*). These Lys residues might be located near the H^+^-releasing residue, and thus, both mutations affect the H^+^-release efficiency. The H^+^-releasing residue should be identified in future studies.

### Putative Cl^−^-releasing pathway

**Figure 9:**
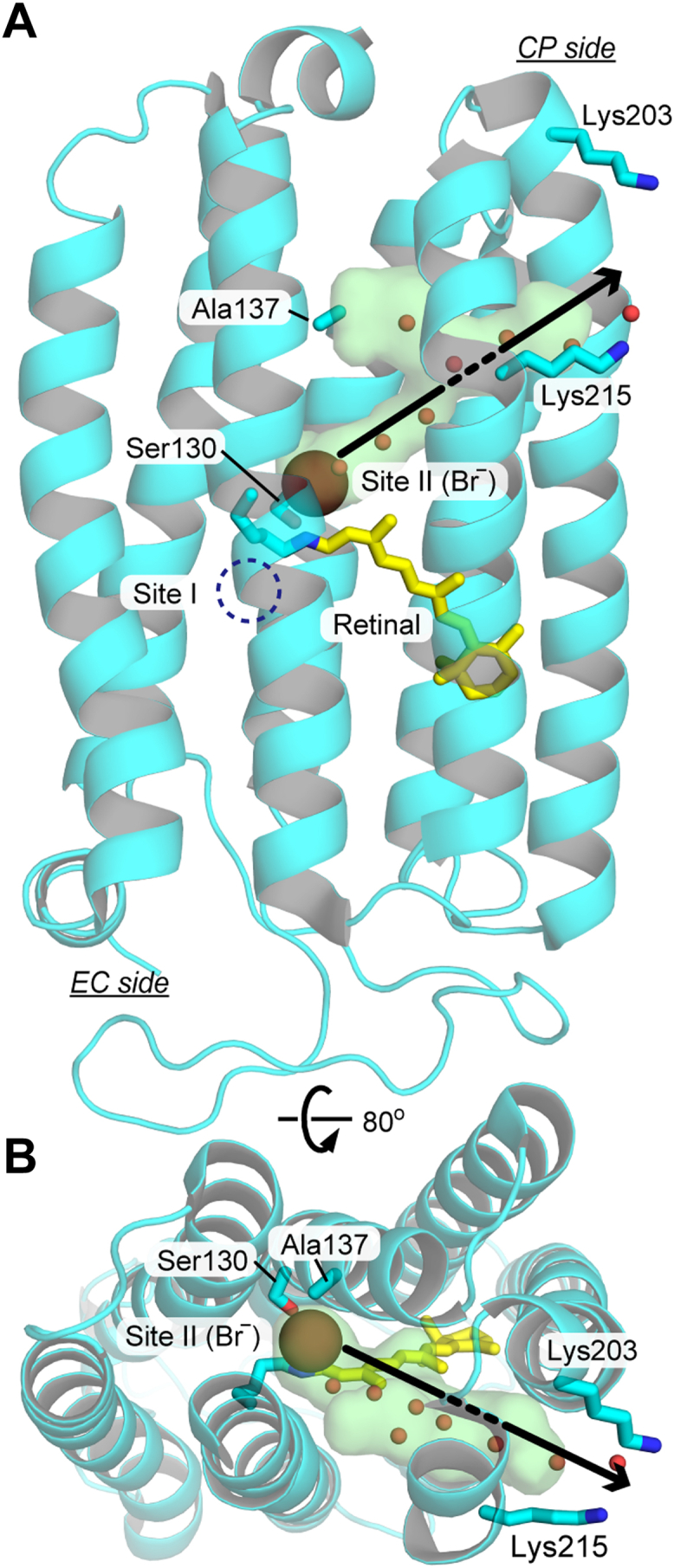
**Putative Cl**^**−**^**-releasing pathway of NpHR**. The crystal structure of N is shown, where seven water molecules (*small red spheres*) are involved in the cavity-created CP side (*transparent green*). This hydrated channel connects Sites II to III, near Lys203 and Lys215. This structure involves Br^−^, which is also the transport substrate of NpHR. The PDB code is 4QRY.

As mentioned above, Site III is probably formed between Lys203 and Lys215 residues located at the protein surface. Thus, this identification also clarifies the Cl^−^-releasing pathway from Site II to the CP medium. Previously, Kouyama *et al.* performed detailed analyses of the crystal structures after photoexcitation and proposed the structures of L1, L2, N, and O intermediates (10). Until the formation of the L2 intermediate, Cl^−^ is located near the original position. During the subsequent N formation, Cl^−^ reaches Site II over the Schiff base region. Besides this Cl^−^ movement, the N structure indicates the formation of a hydrated cavity on the CP side. As shown in Figure 9, the cavity seems to connect Site II and the vicinity of Lys215, suggesting that Cl^−^ is released along the hydrated cavity. However, this pathway is eccentric because its exit is not located on the CP side surface but on the lateral side surface of the protein. For a well-studied H^+^ pump bacteriorhodopsin (BR), H^+^ is captured from the CP medium *via* the Asp96 residue, corresponding to Ala137 of NpHR. This residue is also shown in Figure 9. Thus, for BR, the H^+^-uptake pathway is almost vertical against the membrane surface and significantly different from the hydrated cavity of NpHR. However, our data support this "diagonal" pathway. The overall structure of BR and HR is very similar. However, at least on the CP side, they do not share the substrate transport pathway.

Table S1 summarizes the conservation of Lys203 and Lys215 residues among HRs. Only NpHR conserves positively charged residues at both positions. However, all other HRs conserve a positively charged residue at position 215 of NpHR as Lys or Arg. Thus, for these HRs, one positively charged residue might be sufficient to trap Cl^−^ on their CP surfaces.

### Contradiction to the widely accepted model

Our results are not consistent with the widely accepted model (Figs. 1*B* and 7*A*). There, Cl^−^ release and uptake are assumed to occur during the formation and decay of the O intermediate, respectively. Why did this discrepancy arise? The Cl^−^-selective membrane cannot detect the Cl^−^-transfer reactions at high Cl^−^ concentrations (>100 mM). In contrast, most of the previous studies employed high Cl^−^ concentrations. Thus, although hypothetical, it is possible that the timings of Cl^−^ transfers might change with Cl^−^ concentration. A possible scenario is shown in Fig. S10, which shows putative Cl^−^-transfer reactions at high Cl-concentrations based on our model. As mentioned above, at high Cl^−^ concentration, N intermediates appear in P3 at the quasi-equilibrium state with the O intermediate (15, 23). At around 4 M Cl^−^, the fractions of N and O in the P3 state become nearly equal (15). Thus, 4 M Cl^−^ has been assigned to the dissociation constant of Site II because O is believed to be the Cl^−^ free state. However, we revealed that O is not the Cl^−^-free state, and Site III exists on the Cl^−^-releasing pathway. These two points are involved in Fig. S10. In this figure, Site III is assumed to bind Cl^−^ even in the dark state at high Cl^−^ concentrations and then affect the N-O equilibrium in P3 state. Here, we suppose two N intermediates—N1 and N2. During the formation of N1, Cl^−^ comes to Site II, whereas another Cl^−^ already exists at Site III at high Cl^−^ concentrations. Thus, before Cl^−^ transfer from Sites II to III, Cl^−^ at Site III should be released to the CP medium. N2 formation is associated with this Cl^−^ release. During N2 formation, Site II keeps the Cl^−^, and thus, no spectral change occurs. After that, Cl^−^ moves from Sites II to III during the transition from N2 to O. These three intermediates, N1, N2, and O, might form quasi-equilibrium and simultaneously appear in the P3 state. In this case, N1↔N2 equilibrium should be affected by Cl^−^ binding at Site III in the dark state because it probably slows down the N1→N2 transition. Thus, at high Cl^−^ concentrations, the equilibrium in P3 probably shifts from O to N1 through N2. As mentioned above, a previous study indicated that at 4 M Cl^−^, the fractions of N and O in the P3 state become equal (15). In the present model, the value of 4 M Cl^−^ can be assigned to the dissociation constant of Site III. In Fig. S10, the Cl^−^ release is supposed to associate with the formation of N2, which might simultaneously appear with O due to the fast equilibrium. Thus, Cl^−^ release might be observed simultaneously with the O formation at high Cl^−^ concentration. This timing is the same as predicted in the previous studies (Figs. 1*B* and 7*A*). The present discussion is based on only the data measured at low Cl^−^ concentration. Thus, detailed validation should be necessary in future studies.

## Conclusion

We detected the Cl^−^ release and uptake reactions during a single photocycle of NpHR using the Cl^−^-selective membrane. This observation and the subsequent mutation studies lead to several findings: (1) two additional Cl^−^-binding sites—III and IV—on both the Cl^−^-releasing and Cl^−^-capture pathways; (2) Site III is formed on the protein surface between Lys203 and Lys215 residues, thus, Cl^−^ is not released along the transmembrane helices but released crossing the transmembrane domain diagonally; and (3) Cl^−^ is released from Site III during the O decay and the other Cl^−^ is captured by Site IV during the NpHR′ decay. Despite these findings, several ambiguities remain. Our data suggests that the Cl^−^-binding affinity of Site IV is comparable with that of Site I. Nevertheless, the presence of Site IV has not been proven during the long history of investigation of NpHR. Thus, its existence is still in question. The Cl^−^-transfer reactions at high Cl^−^ concentrations are also unclear due to the limitation of the Cl^−^-selective membrane. Fig. S10 describes our tentative photocycle model at high Cl^−^ concentration. This photocycle and the presence of Site IV should be examined in future studies.

Although the ambiguities remain, it is interesting to consider the functional consequences of the additional Cl^−^-binding sites. Their presence increases the elementary steps for Cl^−^ release and uptake events. Thus, NpHR seems to have a more complex system than previously considered. As the elementary steps increase, a more sophisticated mechanism may be required, but smaller conformational changes may suffice to drive these steps. Larger conformational change requires higher energy costs and accompanies the risk of denaturation. Thus, smaller conformational changes may lead to a faster turnover rate and a longer functional lifetime of NpHR.

## Experimental procedures

### Sample preparation

*E. coli* DH5α was used for DNA manipulation. The expression plasmid of NpHR derived from pET-21c was described previously (27). Mutations were introduced using the QuikChange Site-Directed Mutagenesis Kit (Agilent Technologies). The DNA sequences were confirmed by a standard method. The expression in *E. coli* BL21(DE3) and purification procedures of NpHR were essentially the same as those described previously (28). Lipid reconstitution of the purified NpHR was also performed as previously described (29). For lipids, phosphatidylcholine from egg yolk (Avanti, Alabaster, AL) was used at a protein:lipid molar ratio of 1:50. The reconstituted proteins were collected by centrifugation and then suspended in the appropriate buffer solutions.

### Measurements with a Cl^−^-selective membrane

A polyvinyl chloride (PVC)-based membrane was prepared using 4,5-bis-[N′-(butyl)thioureido]-2,7-di-tert-butyl-9,9-dimethylxanthene (Chloride ionophore IV, Merck), o-nitrophenyl octyl ether (NPOE), and tridodecylmethylammonium chloride (TDDMACl) for the Cl^−^ ionophore, plasticizer, and ion exchanger, respectively. PVC (average polymerization degree, 1050) was purchased from FUJIFILM Wako Chemicals and used without further purification. PVC (80 mg), Cl^−^ ionophore (2.4 mg), NPOE (160 mg), and TDDMACl (12 mg) were dissolved in tetrahydrofuran (2.4 ml). The solution was poured into a Petri dish (5.9 cm^2^ in area), and the solvent was evaporated at room temperature. The resulting membrane was transparent and approximately 0.1–0.2-mm thick.

The experimental setup is schematically shown in Fig. S1*A*. Its details were described previously (19). The electrochemical cell consisted of two Teflon chambers with holes of 5-mm diameter. One of the chambers had another hole for light illumination. A disk with a 10-mm square was cut out from the Cl^−^-selective membrane and inserted between two chambers. For measurements using NpHR, its film was made on the PVC membrane before assembling the photochemical cell. First, the lipid-reconstituted NpHR was suspended in distilled water, and the NpHR concentration was adjusted to 13 μM. The extinction coefficient of 45,500 M^-1^ cm^-1^ at 600 nm was used to calculate the NpHR concentration. This suspension of 50 μl was applied on the Cl^−^-selective membrane and the water component was evaporated under vacuum to make a dry film. This procedure was repeated twice. Consequently, about 1.3-nmol NpHR was deposited on the membrane. This membrane was inserted between two Teflon chambers. The lipid membrane was tightly attached to the PVC membrane, and no detachment was observed during the measurements.

Before all measurements, both chambers were filled with 1-M NaCl solution (1.5-mM HEPES, pH 7.0) and incubated for 2 h to condition the Cl^−^-selective membrane. For the measurements at various Cl^−^ concentrations, only the sample medium was replaced with the buffers containing appropriate concentrations of Cl^−^, which were made by mixing two buffer solutions containing 100-mM NaCl and 33.3-mM Na_2_SO_4_, respectively, so that the ionic strength was kept constant. Both solutions contained 1.5-mM HEPES, and the pH was adjusted to 7.0. For the measurements at various pH, only the sample medium was replaced with a mixed buffer of citric acid and HEPES (10 mM each) containing 1-M NaCl at the appropriate pH. All measurements at various Cl^−^ concentrations and various pH were taken three times. The actinic light for 1-s illumination was prepared using a 150-W Xenon lamp and glass filters (IRA-25S, KL-53, and Y-46; Toshiba). This setup provided green light with a maximum intensity at around 530 nm. The light source for flash excitation was a 5-ns pulse (532 nm and 1 mJ/pulse) of the second harmonic of a Q-switched Nd:YAG laser (Minilite I, Continuum). A homemade amplifier with a 0.033-Hz low-cut filter measured the light-induced potential changes to remove slow baseline drift. For 1-s illumination experiments, the data were obtained 10 times and then averaged to improve the S/N ratio. Similarly, 30 to 50 laser pulses were used for the 5-ns illumination experiments. All measurements were performed at room temperature (∼25 °C).

### Flash-induced absorbance changes and absorption spectra

Transient absorption changes of NpHR during the photocycle were measured with a single absorption kinetic system. The actinic light was a 5-ns laser pulse mentioned above (532 nm and 1 mJ). To improve the S/N ratio, the detected absorption changes were averaged 30 to 50 times. The details of the apparatus were described previously (30). Two different samples were prepared for these measurements. One was a 15% acrylamide gel containing the lipid-reconstituted NpHR. The preparation procedure was previously described (31). This sample had high transparency and enabled the measurements without the precipitation of NpHR. The Cl^−^ concentration was adjusted by immersing the gel into appropriate buffer solutions several times. The other sample was the lipid-reconstituted NpHR deposited on the Cl^−^-selective membrane. The deposition was performed using the same procedure as the membrane potential experiment. This Cl^−^-selective membrane adhered to the inner cell wall of the 10 × 10-mm quartz cuvette, which was then filled with the appropriate buffer solution. All measurements were performed at room temperature (∼25 °C). The buffer solutions were prepared by mixing two solutions containing 100-mM NaCl and 33.3-mM Na_2_SO_4_. Both buffers contained 10-mM HEPES, and the pH was 7.0.

Flash-induced absorbance changes were measured from 400 nm to 710 nm at 10-nm intervals for all samples of acrylamide gels. These data were simultaneously analyzed according to a sequential irreversible model (32), which describes the photocycle with only the forward reactions: P0 → P1 → P2 → P3 → P4 → P0. The term P0 denotes the original dark state, whereas Pi (i = 1–4) denotes the kinetically distinguishable state, which might involve two or more intermediates if back reactions exist among them. For NpHR, four Pi states (i = 1–4) describe the photocycle at any Cl^−^ concentration (15, 23, 27). Using this fitting analysis, we determined the decay time constants of Pi states and their absorption spectra. The latter spectral determinations required independently measured spectra of the P0 state. The absorption spectrum of the lipid-reconstituted NpHR involved a large scattering artifact at a shorter wavelength. Thus, we used the spectrum of detergent-solubilized NpHR in 10-mM HEPES buffer (pH 7.0) containing 100-mM NaCl and 0.05% n-dodecyl-β-D-maltopyranoside (DDM). The details of the analytical procedures were described previously (33).

### Measurements of H^+^ release and uptake reactions of NpHR

These reactions were measured using the indium–tin oxide (ITO) electrode, which acts as a pH electrode with a fast response (24). Measurement procedures were described previously (34). Briefly, the lipid-reconstituted NpHR was deposited on an ITO electrode, and then the electrochemical cell with a bare ITO electrode was constructed, as shown in Fig. S1*B*. The deposited NpHR was activated by a 5-ns laser pulse (532 nm and 1 mJ) at room temperature (∼25 °C). When NpHR exhibits H^+^ transfer reactions, the local pH change occurs near one ITO electrode. The resultant voltage difference between two ITO electrodes was recorded. At each measuring condition, the voltage changes were recorded 30 to 50 times and averaged to improve the S/N ratio. The buffer solutions were the same as those for the potential measurements of Cl^−^-selective membrane.

## Data availability

All of the data supporting the findings of this study are available within the paper and the supporting information.

## Supporting information

This article contains supporting information (24, 26, 35).

## Conflict of interest

The authors declare that they have no conflicts of interest with the contents of this article.
