# Supplementary material for: Direct detection of the chloride release and uptake reactions of Natronomonas pharaonis halorhodopsin
Source: J Biol Chem. 2024 Aug 22;300(9):107712. doi: 10.1016/j.jbc.2024.107712 (PMC11421326; doi:10.1016/j.jbc.2024.107712)
Supplement: Supporting Information [file mmc1.pdf]

Supporting Information

Direct detection of the chloride release and uptake reactions of *Natronomonas pharaonis* halorhodopsin

Chihaya Hamada,[1] Keisuke Murabe,[1] Takashi Tsukamoto,[1,2] and Takashi Kikukawa[1,2,*]

[1]Graduate School of Life Science, Hokkaido University, Sapporo, 060-0810, Japan
[2]Faculty of Advanced Life Science, Hokkaido University, Sapporo, 060-0810, Japan

[*] Corresponding author
Dr. Takashi Kikukawa, E-mail: kikukawa@sci.hokudai.ac.jp.

Table of Contents

1. Supplemental Results....................................S3

1-1. Detection of the H^+-transfer reactions by NpHR....................................S3

1-2. Removal of the contribution of "nonpumping photocycle" from the measured absorbance changes...S3

2. Supplemental Discussion....................................S5

2-1. Cl^--concentration dependence of the coefficient f_4....................................S5

2-2. Discrepancy from the dark state structure....................................S6

3. Supplemental Figures and Tables....................................S7

Figure S1. Schematic illustrations of the photochemical cells used in this study....................................S7

Figure S2. Constant light-induced potential changes in the Cl^--selective membrane....................................S8

Figure S3. Comparison of the time course of membrane potential and absorbance changes....................................S9

Figure S4. Flash-induced H^+-transfer reactions....................................S10

Figure S5. Estimation of flash-induced absorbance changes derived from Cl^--pumping NpHR....................................S11

Figure S6. Flash-induced absorbance changes derived from the Cl^--pumping NpHR....................................S12

Figure S7. Decay time constants of Pi (i = 1–4) states....................................S13

Figure S8. The time course of intermediate accumulations and membrane potential changes....................................S14

Figure S9. Cl^--concentration dependences of f_i (i = 1–4) values....................................S15

Figure S10. Putative Cl^--transfer reactions of NpHR at high Cl^- concentrations....................................S16

Table S1. Comparison among HRs for residues corresponding to K203 and K215 in NpHR....................................S17

1. Supplemental Results

1-1. Detection of the H^+-transfer reactions by NpHR

As shown in Fig. 2B, the potential of the Cl^--selective membrane has a positive slope against pH. Thus, the negative potential changes in upper panels of Fig. 4 might reflect the decrease in pH, that is, the photolyzed NpHR might exhibit H^+ release first followed by the uptake. We tested this hypothesis using the ITO electrode, which is a transparent and pH-sensitive electrode with a fast response (24). Herein, we used an electrochemical cell in Fig. S1B. The lipid-reconstituted NpHR was deposited on the working electrode and then activated by the laser pulse. Time-dependent voltage changes are plotted in Fig. S4A. In this experiment, the positive signal indicates the pH decrease around the working electrode (24), that is, the photolyzed NpHR surely exhibits H^+ release first, followed by uptake. The detected signals are small but appear around 2 ms after the flash, which is almost at the same time as the negative potential change of the Cl^--selective membrane (Fig. 4).

The H^+ release from the photolyzed NpHR was previously suggested by an FTIR study, where the H^+ source was assigned to Glu234 residue around the EC surface (Fig. 1A) (35). Thus, we also performed ITO experiments for the E234Q NpHR mutant. The results are shown in Fig. S4B, where the H^+-release signals still appear. Thus, Glu234 is not the source of H^+.

1-2. Removal of the contribution of "nonpumping photocycle" from the measured absorbance changes

Figure S5A shows the three-wavelength data of the nonpumping photocycle, which are measured at 0 mM Cl^-. The initial absorbance change at 500 nm is almost constant until 0.1 ms. This value gradually increases with the increase in the Cl^- concentration, reflecting the increase of Cl^--bound NpHR ("500 nm" traces in Fig. 4). Thus, using the signals at 500 nm, we estimated the fractions of Cl^--bound NpHR. In Fig. S5B, the net increases in signal amplitudes ($\Delta\Delta A_{500}$) are plotted against Cl^- concentration with the filled circles. The solid line is the best-fit curve using the following equation:

$$A_{\max} \cdot f_{\mathrm{Cl}} = A_{\max} \cdot [\mathrm{Cl}^-] / ([\mathrm{Cl}^-] + K_{\mathrm{d}}) \qquad \text{(S1)},$$

where $A_{\max}$ and $[Cl^-]$ stand for the maximum amplitude of the plot and Cl^- concentration, respectively, and f_{Cl} represents the fraction of the Cl^--bound NpHR. The determined K_{d} is 7.4 mM, whose value roughly matches the K_{d} value determined from the Cl^--induced spectral shift of the unphotolyzed state. This spectral shift is shown in Fig. S5C. Upon binding of Cl^-, the $\lambda_{\max}$ shifts to a shorter wavelength and finally reaches 580 nm. The absorbance increase at 580 nm (ΔA_{580}) was calculated and then plotted in Fig. S5B

with the open circles. The best-fit result (broken line) was obtained with a K_d of 3.6 mM. Thus, both plots surely reflect the Cl^--dependent increase of f_{Cl}. Herein, we used the K_d from the $\Delta\Delta A_{500}$ to calculate the fraction of Cl^--free NpHR (f_{free}) as follows:

$$f_{free} = 1 - f_{Cl} = K_d \,/\, ([Cl^-] + K_d) \qquad \text{(S2)}.$$

Next, we removed the contribution of the nonpumping photocycle from the measured absorbance changes. Here, we simply calculated the following subtractions for all wavelength data:

$$\Delta\Delta A_{Cl}(t, \lambda) = \Delta A_{Cl}(t, \lambda) - f_{free} \cdot \Delta A_{free}(t, \lambda) \qquad \text{(S3)},$$

where $\Delta A_{Cl}(t, \lambda)$ and $\Delta A_{free}(t, \lambda)$ are the measured flash-induced absorbance changes in the presence and absence of Cl^-, respectively. The remaining absorbance change $\Delta\Delta A_{Cl}(t, \lambda)$ originates from only NpHR exhibiting Cl^--pumping photocycle. The typical example of this calculation is shown in Fig. S5D, which involves three-wavelength data at 3-mM Cl^- before ($\Delta A_{Cl}(t, \lambda)$, thin lines) and after the subtraction ($\Delta\Delta A_{Cl}(t, \lambda)$, thick lines). Corresponding figures at other Cl^- concentrations are shown in Fig. S6. We performed the global fitting analyses against the dataset of $\Delta\Delta A_{Cl}(t, \lambda)$.

2. Supplemental Discussion

2-1. Cl^--concentration dependence of the coefficient f_4

$P_4(t)$ is proportional to the amount of NpHR′. Thus, the Cl^- concentration change by NpHR′ ($\Delta[Cl^-](t)$) is expressed as follows:

$$\Delta[Cl^-](t) = k \cdot P_4(t) \qquad \text{(S4)}$$

where k is the proportionality coefficient. According to Eq. 2, the corresponding potential change, $\Delta\Psi_{Cl}(t)$, is expressed as follows:

$$\Delta\Psi_{Cl}(t)\ [\text{mV}] = 54.5 \log(1 + \Delta[Cl^-](t) / [Cl^-])$$
$$\approx\ 54.5 \cdot \Delta[Cl^-](t) / [Cl^-] = 54.5 \cdot k \cdot P_4(t) / [Cl^-] \qquad \text{(S5)}$$

where Eq. S4 is used. The potential change of $\Delta\Psi_{Cl}(t)$ corresponds to $f_4{\cdot}P_4(t)$. Therefore, the deformation of Eq. S5 takes the following form:

$$\Delta\Psi_{Cl}(t)\ [\text{mV}] = f_4 \cdot P_4(t) = 54.5 \cdot k \cdot P_4(t) / [Cl^-] \qquad \text{(S6)}$$

Thus, f_4 is described as follows:

$$f_4 = 54.5 \cdot k / [Cl^-] = k' / [Cl^-] \qquad \text{(S7)}$$

where k' is also the proportionality coefficient. Consequently, f_4 is inversely proportional to the Cl^- concentration. This relationship actually appears in Fig. S9. The value of 54.5 included in Eq. S7 comes from the slope of the plot in Fig. 2A (54.5 mV/decade). However, this slope was smaller (19.1 mV/decade) below 3 mM Cl^-. Thus, f_4 at 1 mM Cl^- cannot be directly compared to f_4 values at higher Cl^- concentrations. In Fig. S9, f_4 at 1 mM Cl^- is plotted but is not involved in the fitting analysis by Eq. S7.

2-2. Discrepancy in the dark state structure

Regarding Site IV, we failed to identify its position through the mutation experiments. Two mutants of Arg22 and Arg176 could not be prepared due to their negligible expression in *E. coli*. Thus, either Arg sidechain might form Site IV. In contrast, the presence of Site IV is not consistent with the crystal structure of the dark state because, on its EC side, Cl^- is only observed at Site I (26). As shown in Fig. 4, the Cl^--

transfer timings are maintained even at a low Cl^- concentration of 1 mM, indicating that both Sites I and IV bind Cl^- at 1-mM Cl^-. Thus, the Cl^--binding affinity of Site IV is at least comparable with that of Site I. Why is Cl^- binding at Site IV not observed in the crystal structure despite the binding at Site I being observed? One explanation might be Cl^- binding after photoexcitation. If this uptake by Site IV occurs at a very fast rate, the phase of potential decrease cannot be observed. This fast Cl^- uptake seems to correspond to the negative potential change observed at earlier time ranges (Fig. 4, upper panels). However, as mentioned above, the early potential change does not disappear even at 100-mM Cl^-. Thus, at present, we could not assign this potential change to the Cl^--transfer reaction. Thus, the location of Site IV should be further investigated in future studies.

3. Supplemental Figures

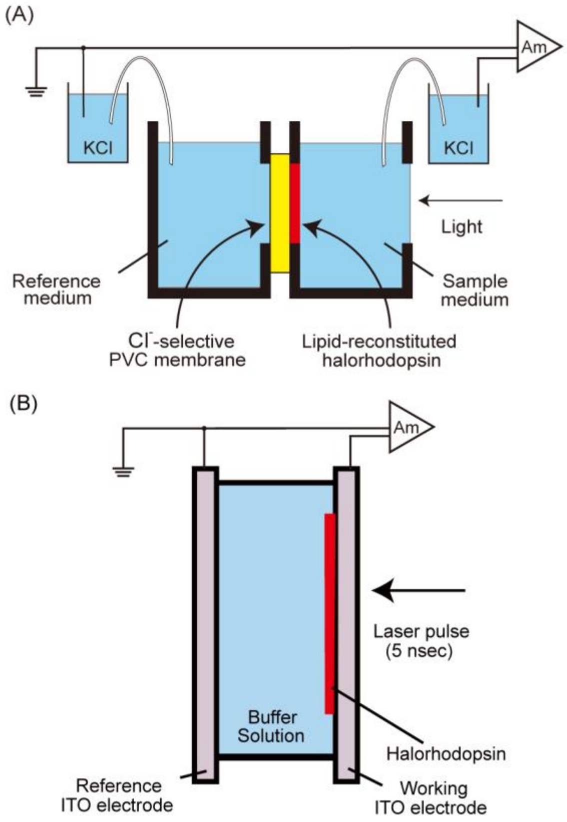

Figure S1. Schematic illustrations of the photochemical cells used in this study. (A) The cell is equipped with a Cl^--selective membrane for measuring Cl^--transfer reactions of NpHR. Am denotes the voltage amplifier. (B) The cell is equipped with two ITO electrodes for measuring H^+-transfer reactions of NpHR.

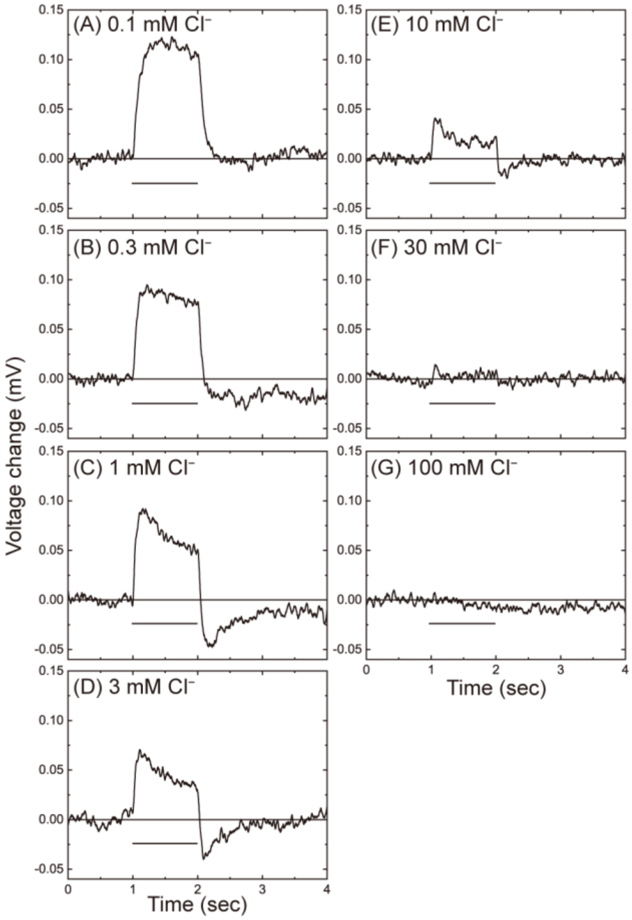

Figure S2. Constant light-induced potential changes of the Cl^--selective membrane. We deposited the lipid-reconstituted NpHR on the membrane surface facing the sample chamber. The horizontal bar indicates the duration of illumination. The data in panels C and G are the same as those in Fig. 3A and B, respectively.

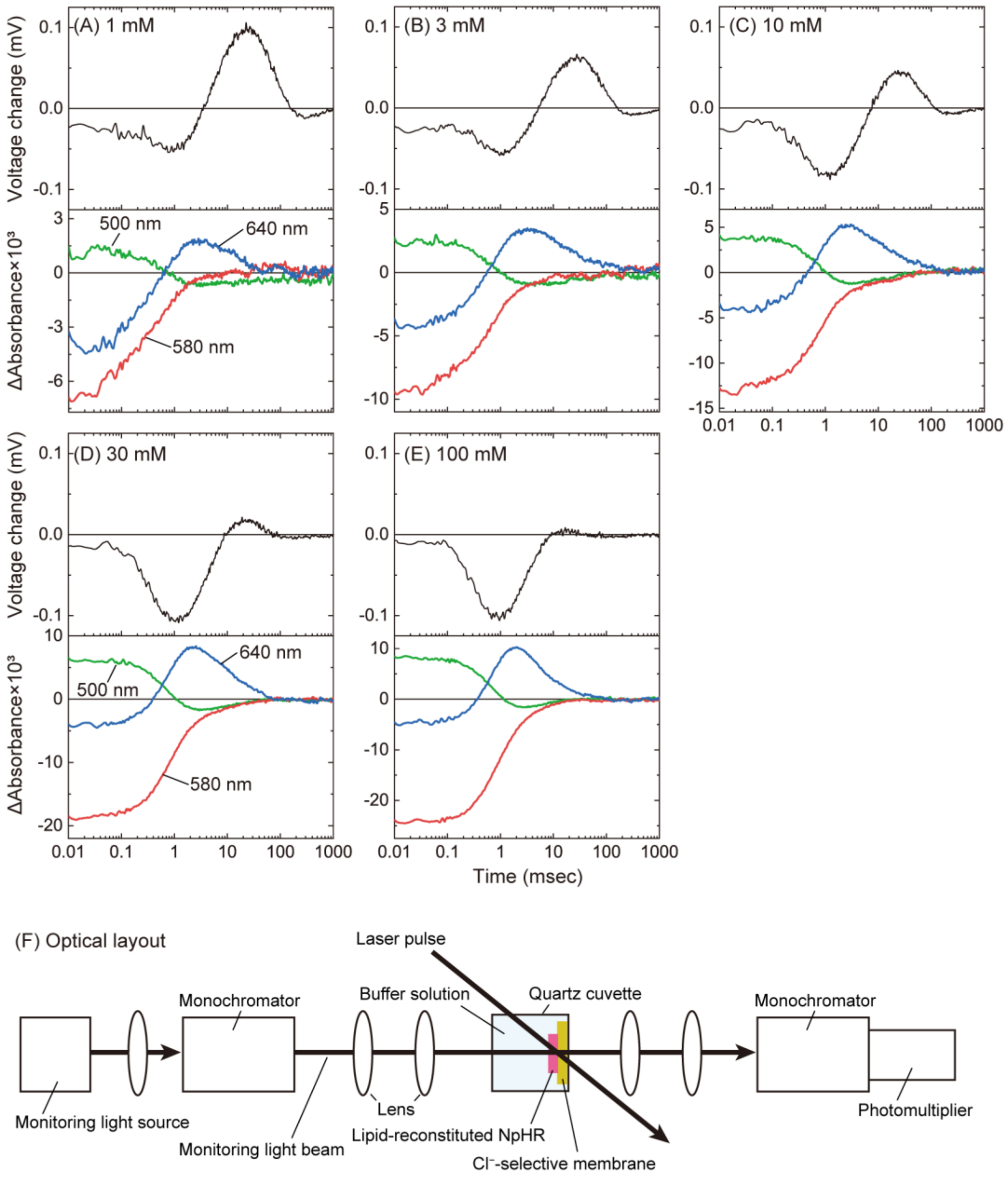

Figure S3. Comparison of the time course of membrane potential and absorbance changes. The upper panels of A–E display the flash-induced potential changes, which are the same data shown in Fig. 4. They were measured for the lipid-reconstituted NpHR deposited on the Cl^--selective membrane. We used the same sample to measure the flash-induced absorbance changes in the lower panels. The optical layout is shown in F, where the Cl^--selective membrane with NpHR adhered to the inner cell wall of the quartz cuvette filled with an appropriate buffer solution.

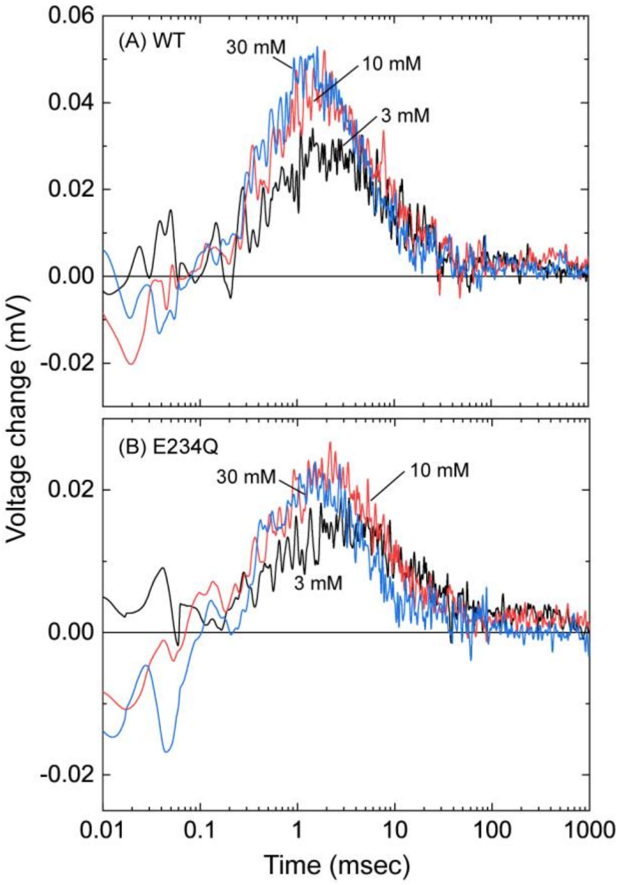

Figure S4. Flash-induced H^+-transfer reactions. The time-dependent voltage differences between two ITO electrodes were plotted for the (A) wild-type NpHR and (B) E234Q mutant. The Cl^- concentrations are indicated in the panels.

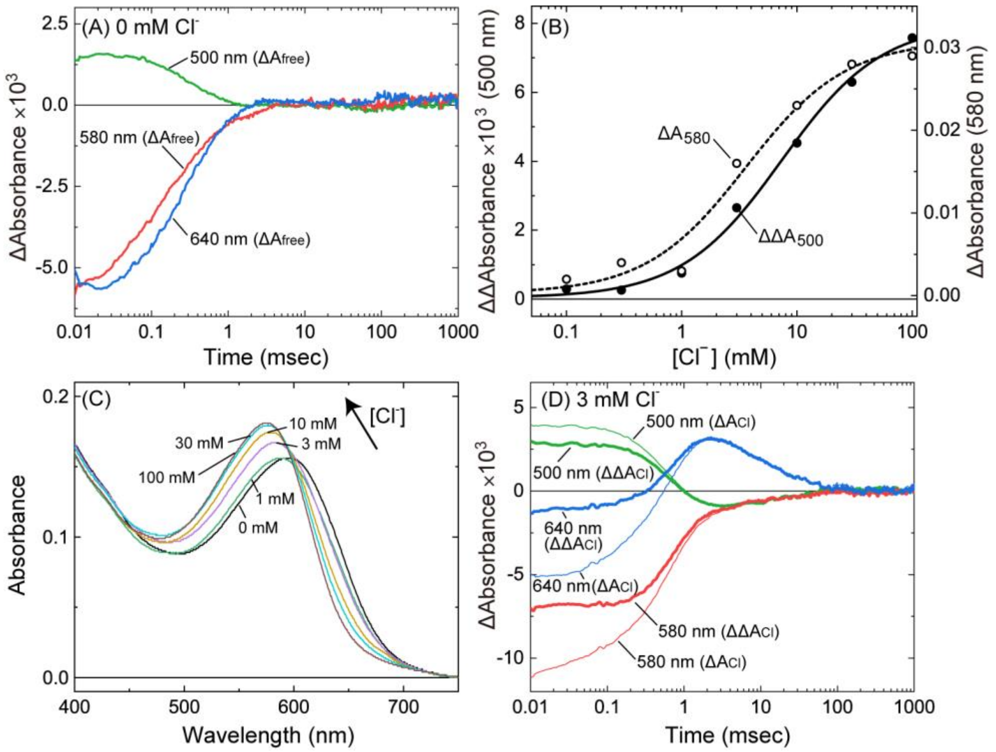

Figure S5. Estimation of flash-induced absorbance changes derived from Cl⁻-pumping NpHR. (A) Flash-induced absorbance changes measured at 0-mM Cl⁻ corresponding to ΔA_{free} (t, λ). Only data at three wavelengths are shown. (B) Estimation of the dissociation constant of the unphotolyzed NpHR for Cl⁻. Filled circles indicate the Cl⁻-dependent increase of the flash-induced signal at 500 nm. The absorbance changes at 0.01 ms were selected, and the differences ($\Delta\Delta A_{500}$) from the value at 0 mM Cl⁻were plotted using the left axis. Open circles were derived from the Cl⁻-dependent absorption spectral shift of the unphotolyzed NpHR, whose raw spectra are plotted in (C). The absorbance increases at 580 nm (ΔA_{580}) were calculated and plotted in (B) by using the right axis. The solid and broken lines are the best-fit curves using Eq. S1. (D) Estimation results for the data at 3-mM Cl⁻. The thin and thick lines represent the raw data ($\Delta A_{\mathrm{Cl}}(t, \lambda)$) and the estimated absorption changes by the Cl⁻-pumping NpHR ($\Delta\Delta A_{\mathrm{Cl}}(t, \lambda)$), respectively. Only data at three wavelengths are shown. For details, see the Supplemental Results 1-2.

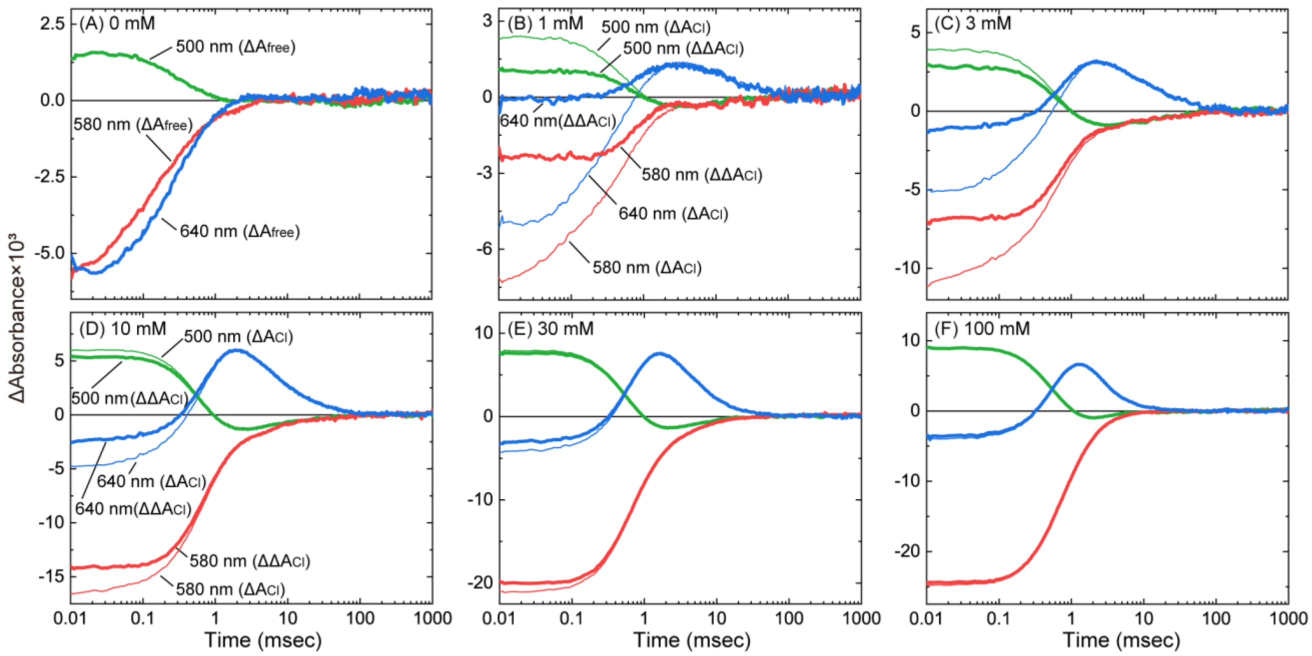

Figure S6. Flash-induced absorbance changes derived from the Cl⁻-pumping NpHR. The data in (A) for 0-mM Cl⁻ and (D) for 3-mM Cl⁻ are the same as those in Fig. S5A and C, respectively. Panels (B–F) involve both the raw data (thin lines, $\Delta A_{Cl}(t, \lambda)$) and the estimated components derived from the Cl⁻-pumping NpHR (thick lines, $\Delta\Delta A_{Cl}(t, \lambda)$). In panel (F), two components are almost overlapped because at 100-mM Cl⁻ most NpHR binds Cl⁻ in the dark state and exhibits a Cl⁻-pumping photocycle. The raw data (thin lines) in (B)–(F) are the same with those in lower panels of Fig. 4. The calculated absorbance changes (thick lines) in (B)–(F) are also plotted in upper panels of Fig. 5 and Fig. S8 for further analysis. For details, see the Supplemental Results 1-2.

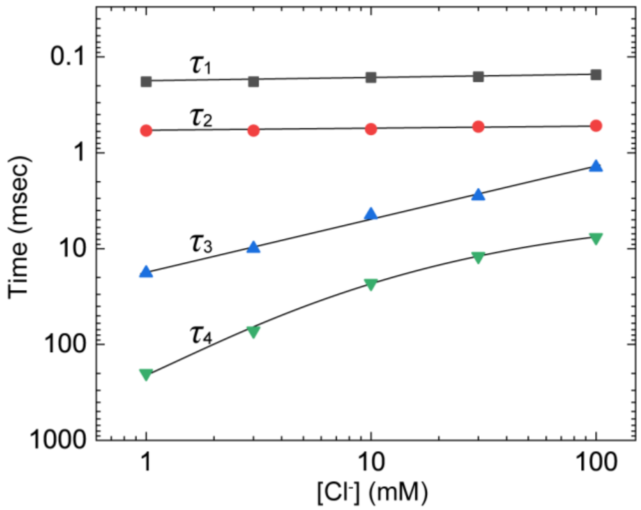

Figure S7. Decay time constants of Pi (i = 1–4) states.

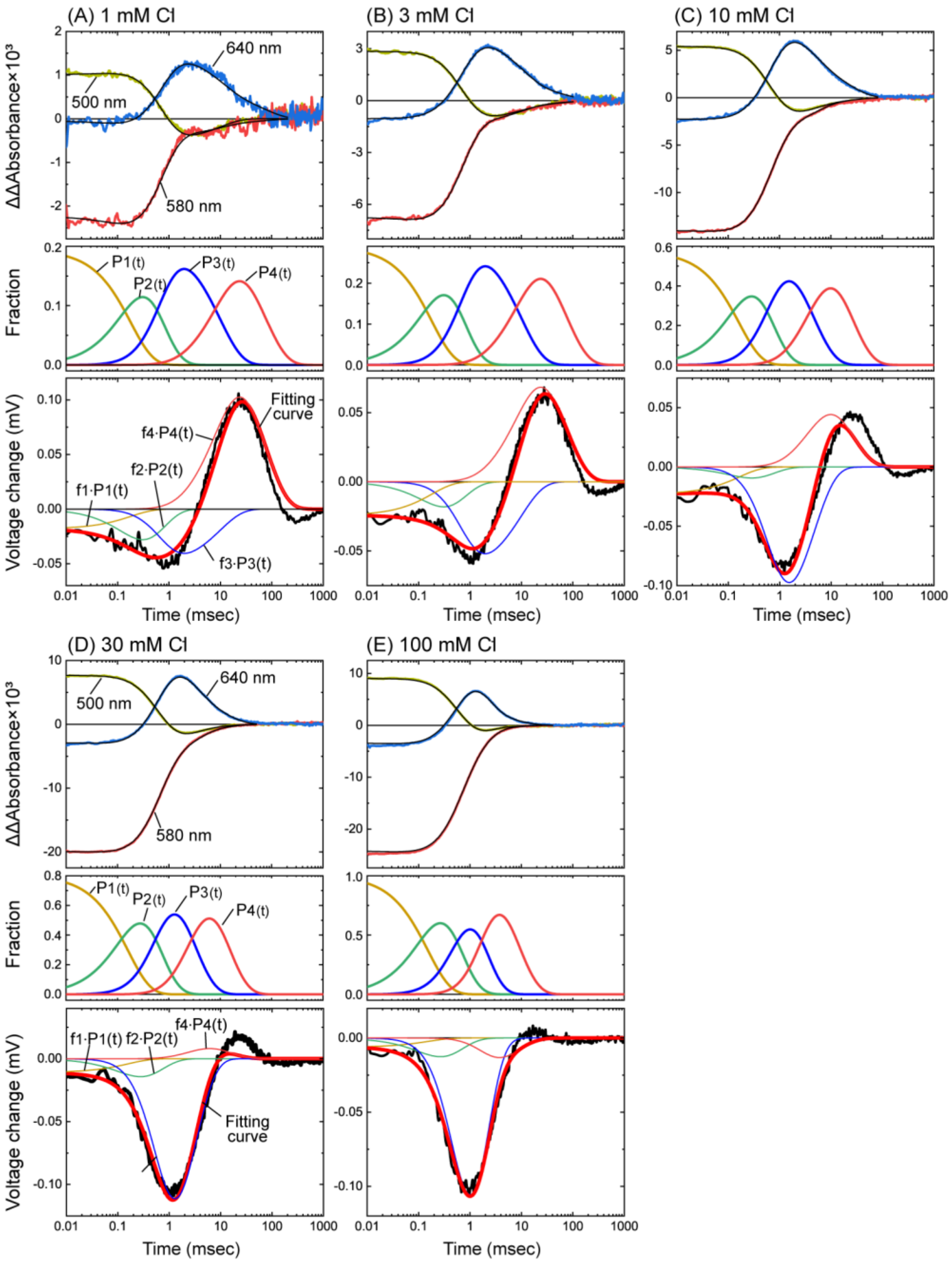

Figure S8. The time course of intermediate accumulations and membrane potential changes. The differences from Fig. 5 are only the data in the bottom panels. The simulation curves (thick red lines) in those panels are the best-fit results of the membrane potential changes by the fitting software. Their components, $f_i \cdot P_i(t)$ (i = 1–4) (thin lines), are also different from those in Fig. 5. For details, see the text.

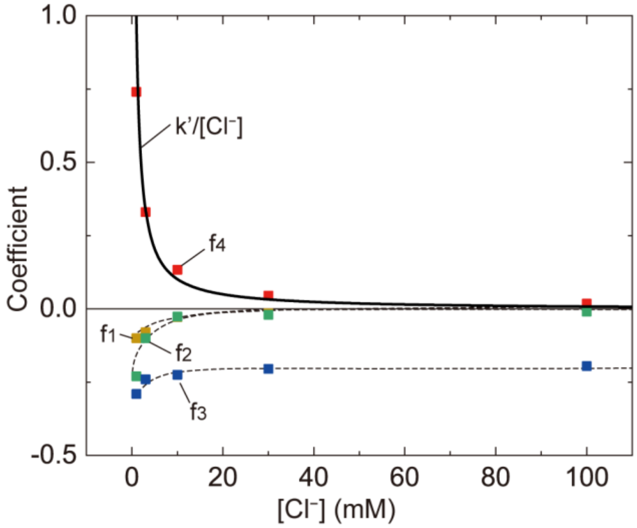

Figure S9. Cl^- concentration dependences of f_i (i = 1–4) values. The broken lines for f_1–f_3 are shown for better comprehension. The thick solid line for f_4 is the best-fit curve derived using Eq. S7. For details, see the Supplemental Discussion 2-1.

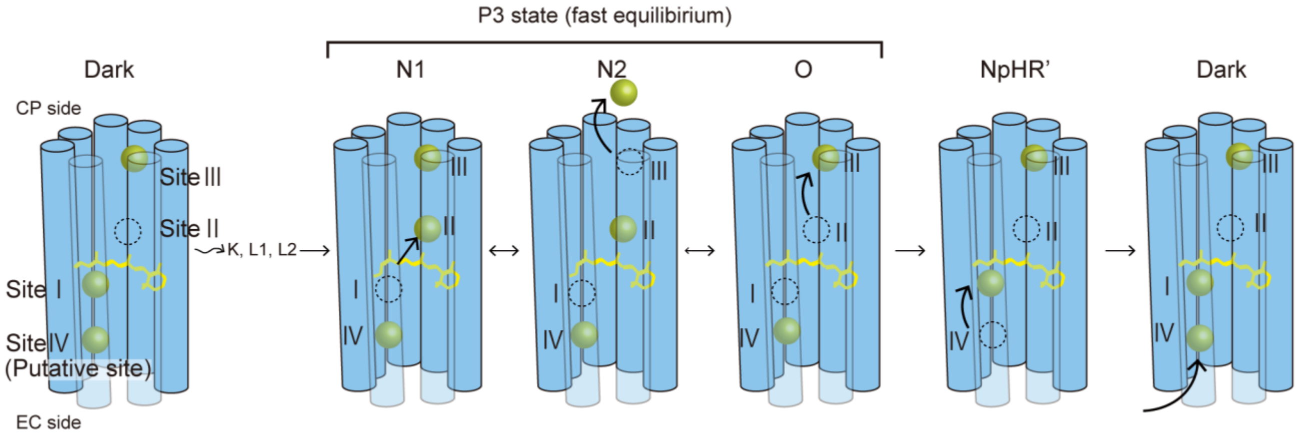

Figure S10. Putative Cl⁻-transfer reactions of NpHR at high Cl⁻ concentrations. Site III is supposed to bind Cl⁻ even in the dark state. Before forming the O intermediate, Cl⁻ should be released into the CP medium. The two N intermediates, N1 and N2, reflect the states before and after the Cl⁻ release. The N1 to N2 transition is probably slowed down by the Cl⁻ binding at Site III. Resultantly, at high Cl⁻ concentrations, the equilibrium in the P3 state shifts from O to N1 through N2. For details, see main text.

Table S1. Comparison among HRs for residues corresponding to K203 and K215 in NpHR

Species	Accession ID	203	215
Natronomonas pharaonis (NpHR)	P15647	**K**	**K**
Halobacterium salinarum	CAP13054	**S**	**R**
Halobacterium salinarum shark	BAA07822	**E**	**K**
Halobacterium salinarum port	Q48315	**E**	**K**
Haloarcula vallismortis	BAA06679	**E**	**K**
Halobacterium sp. SG1	CAA49773	**E**	**K**
Haloterrigena sp. Arg-4	BAA75201	**E**	**R**
Halorubrum sodomense	BAA75202	**E**	**K**
